# Survival Prediction Based on Compound Covariate under Cox Proportional Hazard Models

**DOI:** 10.1371/journal.pone.0047627

**Published:** 2012-10-24

**Authors:** Takeshi Emura, Yi-Hau Chen, Hsuan-Yu Chen

**Affiliations:** Institute of Statistical Science, Academia Sinica, Nankang, Taipei, Taiwan; Harvard School of Public Health, United States of America

## Abstract

Survival prediction from a large number of covariates is a current focus of statistical and medical research. In this paper, we study a methodology known as the compound covariate prediction performed under univariate Cox proportional hazard models. We demonstrate via simulations and real data analysis that the compound covariate method generally competes well with ridge regression and Lasso methods, both already well-studied methods for predicting survival outcomes with a large number of covariates. Furthermore, we develop a refinement of the compound covariate method by incorporating likelihood information from multivariate Cox models. The new proposal is an adaptive method that borrows information contained in both the univariate and multivariate Cox regression estimators. We show that the new proposal has a theoretical justification from a statistical large sample theory and is naturally interpreted as a shrinkage-type estimator, a popular class of estimators in statistical literature. Two datasets, the primary biliary cirrhosis of the liver data and the non-small-cell lung cancer data, are used for illustration. The proposed method is implemented in R package “compound.Cox” available in CRAN at http://cran.r-project.org/.

## Introduction

Predicting survival outcomes in the presence of a large number of covariates has received much attention in the recent decade. The prominent motivation for this comes from predictions of patient survival based on gene expression profiles. For example, gene expression profiles have been used to improve the prediction power of the clinical outcomes for breast cancer patients [1], [2], [3], [4] and lung cancer patients [5], [6], [7]. Utilizing gene profiles, van’t Veer et al. [3] provided a criterion for selecting patients who would benefit from adjuvant therapy, which reduces patients’ risks over traditional guidelines based on histological and clinical characteristics. Chen et al. [6] examined 672 gene profiles for non-small-cell lung cancer patients to identify a gene signature closely related to survival. Even without gene expression profiles, patients data often include a large number of clinical, serologic and histologic characteristics. Hence, it is of interest to efficiently utilize a large number of covariates to predict clinical outcomes.

A statistical challenge arises if the number of covariates *p* is large relative to the number of individuals *n*. The problem becomes further involved with the presence of censoring. The standard regression techniques in the presence of censoring, including the Cox regression analysis [8], fail to provide a satisfactory result.

Two types of strategies have been commonly used to perform survival prediction with a panel of covariate data. The first strategy is to select subsets of covariates by univariate survival analyses [1], [6] or various clustering algorithms [9]. Then, one can apply standard methods for prediction. The second strategy for resolving high-dimensionality utilizes some penalizing schemes on the Cox regression analysis. In particular, the Lasso [10], [11], [12] and ridge regression [13], [14] are obtained by penalizing the Cox’s partial likelihood function with 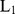 and 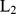 penalties, respectively. The two types of penalization yield *p* regression coefficients that are shrunk toward zero.

In this paper, we study a methodology known as the compound covariate prediction. The compound covariate prediction method is based on a linear combination of the univariate Cox regression estimates and has been previously used in medical studies with microarrays [5], [6], [15], [16]. However, few papers have investigated its statistical properties and comparative performance with other methods. For instance, recent comparative studies of Bovelstad et al. [17], van Wieringen et al. [18], and Bovelstad and Borgan [19] have all demonstrated that ridge regression has the overall best predictive performance among many well-known survival prediction methods, including univariate selection, forward selection, Lasso, principal components, supervised principal components, partial least squares, random forests, etc., but excluding the compound covariate method. Additionally, the compound covariate prediction can be a powerful method even for more traditional survival data that may not involve microarrays, as we will see in the analysis of the primary biliary cirrhosis of the liver data. Hence, the first objective of this paper is to study the statistical properties and comparative performance of the compound covariate method, in order to fill a gap in the current literature and highlight the competitive performance of the compound covariate method with other methods.

The second objective of this paper is to develop a new statistical methodology that refines the compound covariate method. This methodology aims to incorporate the combined predictive information of covariates into a compound covariate predictor by forming a mixture of multivariate and univariate Cox partial likelihoods. Such a method is shown to have a theoretical justification under a statistical large sample theory, and is naturally interpreted as a shrinkage-type estimator, a popular class of estimators in statistical literature.

We also compare the compound covariate and the newly proposed methods with the benchmark methods of ridge regression and Lasso analyses via Monte Carlo simulations and real data analysis. The primary biliary cirrhosis of the liver data and the non-small-cell lung cancer data are used for illustration. All the numerical performances of the methods are evaluated via cross-validated schemes.

## Methods

### Existing Methods

To facilitate the subsequent discussions, we shall introduce existing methods for predicting survival outcomes. Let 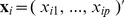 be a 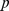-dimensional vector of covariates from individual 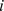. We observe 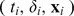, where 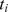 is either survival or censoring time, and 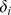 satisfies 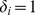 if 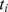 is survival time and 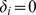 otherwise. In the Cox regression [8], the hazard function for individual 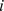 is modeled as
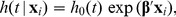
(1)


where 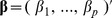 are unknown coefficients and 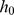 is an unknown baseline hazard function. Let 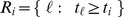 be the risk set that contains individuals who still survive at time 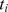. The regression estimate is obtained by maximizing the partial likelihood given as
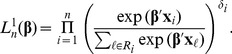
(2)


When the dimension *p* is large relative to the sample size *n*, the maximum of 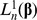 is not uniquely determined.

An intuitive and widely used approach to resolve high-dimensionality is based on the *univariate selection*. As the initial step, a Cox regression based on the univariate model 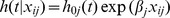, or a log-rank test between the high and low covariate groups, is performed for each 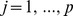, one-by-one. Then one picks out a subset of covariates that have low P-values from the univariate analysis (e.g., Jenssen et al. [1]). The top 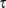 covariates with lowest P-values are then included in a multivariate Cox regression, where the number 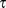 can be determined by cross-validation and/or biological consideration. Although the univariate selection is easy to implement, the process of selecting covariates is solely based on the marginal significance, and hence there is no guarantee that the resultant multivariate model achieves an accurate prediction.

A more sophisticated approach to resolve high-dimensionality is to utilize the 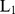 penalized partial likelihood
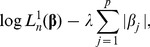
(3)


or the 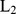 penalized partial likelihood
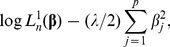
(4)


where 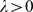 is the tuning (shrinkage) parameter. The two methods shrink the coefficients to zero. The estimator resulting from equation (3) is called the Lasso [10], [11], [12]. An important feature of the Lasso is that many coefficients will be estimated exactly as zero. This implies that the Lasso can be used as a variable selection tool for a parsimonious prediction model. On the other hand, the estimation based on equation (4) is called ridge regression [13], [14], which results in *p* non-zero coefficient estimates. Therefore, unlike the Lasso, the prediction model from ridge regression uses all the covariates. The tuning parameter 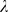 can be obtained empirically by a cross-validation criterion proposed by Verweij and van Houwelingen [20]. Both the Lasso and ridge regression methods are implemented through the R package “penalized” [21].

There are a number of other methods available to handle high-dimensional covariates, including the forward stepwise selection, principal components, supervised principal components, Lasso principal components, partial least squares regression, and tree-based methods, etc.; refer to Witten and Tibshirani [22] for an excellent summary. Bovelstad et al. [17], van Wieringen et al. [18], and Bovelstad and Borgan [19] systematically compared these methods and concluded that ridge regression has the best overall performance for survival prediction. However, the compound covariate method has not been included in these comparative studies.

### Compound Covariate Prediction

For a future subject with a covariate vector 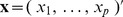, the survival prediction can be made by the prognostic index (PI) defined as 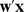, where 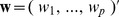 is a vector of weights. Typically, 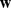 is determined by the dataset 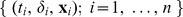 and is chosen so that 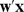 is associated with the subject’s survival. When *p* is small relative to *n*, the multivariate Cox’s partial likelihood estimator maximizing equation (2) can be used for 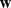. Alternatively, one can set 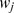 to be the estimated regression coefficient for 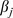 by fitting the univariate Cox model 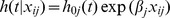, for each 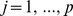, one-by-one. This prediction method is called *the compound covariate prediction* [23] and it is applicable even when *p> n*. The method has been shown to be useful in medical studies with microarrays as a convenient and powerful tool for survival prediction [5], [6], [15], [16]. Note that even when *p*< *n*, where a multivariate Cox regression is applicable, the compound covariate prediction may further improve predictive power. We will demonstrate this aspect through the analysis of the primary biliary cirrhosis of the liver data.

### Refinement of the Compound Covariate Method

The construction of the compound covariate predictor is purely based on the univariate (marginal) likelihood functions. This methodology may be further improved by incorporating the combined predictive information of covariates into the compound covariate predictor. Here we propose a mixture of the multivariate and univariate (marginal) likelihoods. For each covariate 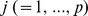, the univariate Cox regression estimator for 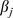 is obtained by maximizing
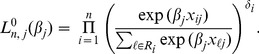
(5)


**Figure 1:**
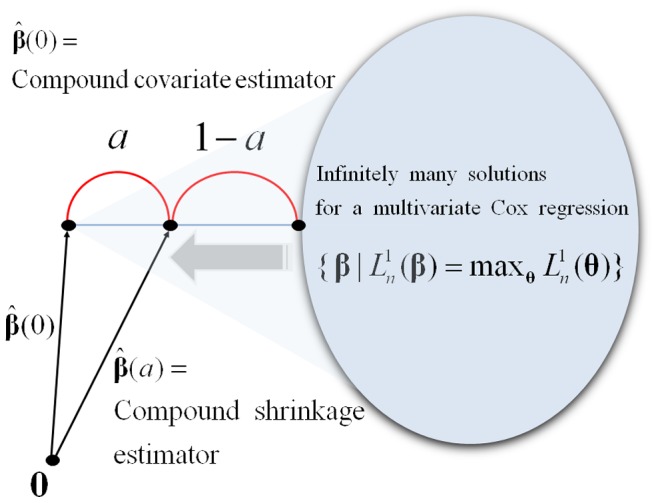
The proposed shrinkage scheme applied for the compound covariate method.

We combine the likelihoods in equation (5) over all 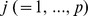, namely,
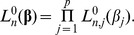



Note that the maximizer of 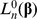 is found as the set of the 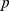 univariate Cox regression estimates even when 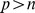, and hence 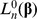 adapts easily to high-dimensionality. On the other hand, 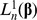 does not have a unique solution when 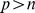, although it potentially contains the combined predictive information of covariates. To gain an adequate compromise between 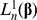 and 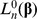, we consider a mixture log-likelihood
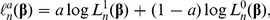
(6)


where 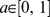 is the tuning (shrinkage) parameter. For a fixed 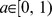, the maximizer of equation (6) is denoted by 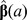. We will call 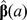 *the compound shrinkage estimator*, and 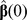 *the compound covariate estimator*, which is a special case of 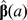 at 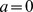. The compound shrinkage predictor 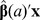 can thus be viewed as a generalization of the compound covariate predictor 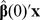, with a larger 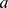 leading to a larger degree of multivariate likelihood information (Figure 1). It will be seen that the value of 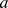 can be empirically estimated by cross-validation.

The idea of the compound shrinkage as a mixture of the multivariate and univariate likelihoods is closely related to a “shrinkage” scheme in statistical literature. This has the effect of reducing (shrinking) the infinite dimensional solution space of the multivariate likelihood equations toward the unique nearest point of 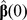 as demonstrated in Figure 1. Here, 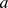 = 0 stands for the maximal shrinkage and 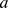 = 1 for no shrinkage.

### Choosing the Shrinkage Parameter by Cross Validation

The shrinkage parameter 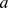 in equation (6) should be chosen so that the predictive power of 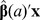 is maximized. For this purpose, we adopt a cross-validation criterion based on partial likelihood [20]. To perform a 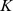-fold cross validation, we first divide 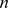 individuals into 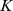 groups of about equal sample sizes, and label them as 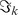 for 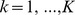. The maximizer of equation (6) based on all individuals not in 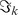 is calculated and denoted by 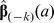. Repeat this process for 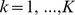, and the cross-validation criterion is
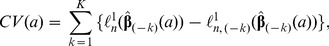
(7)


where 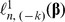 is the log-partial likelihood based on all individuals not in 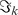. Finally, we find 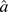 that maximizes equation (7). The numbers 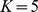 or 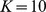 are used commonly when *n* or *p* is large [16], [17], [24]. Since the resultant estimators 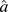 and 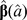 are fairly robust against the choice of 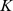 in our simulations, we recommend 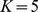 for computational simplicity.

## Numerical Results

### Evaluation Criteria

We first revisit the three measures for prediction accuracy proposed by Bovelstad et al. [17]. Let 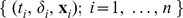 be a training dataset and 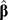 an estimator obtained from the training dataset, and let 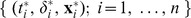 be a test dataset.

1) *Log-rank test (LR-test):* Subject 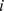 in the test dataset is categorized in the good (poor) prognosis group if 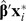 is below (above) the median of 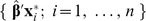. The P-value for a log-rank test performed in the test dataset for comparing survival times in the two groups represents prediction performance. Smaller P-value corresponds to better prediction ability.

2) *Cox regression test (Cox-test):* By treating 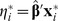 as a covariate, the Cox model 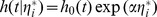 is fitted to 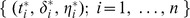. The P-value for testing the hypothesis 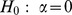 represents a measure of prediction ability. Smaller P-value corresponds to better prediction ability.

3) *Deviance (Devi)*: Let 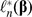 be the log-partial likelihood function calculated from the test dataset. The deviance 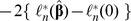 measures how the model with 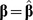 improves the null model with 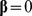 in terms of goodness-of-fit in the test dataset. Smaller deviance corresponds to better prediction ability.

We further consider the 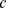-index proposed by Harrell et al. [25], [26], which is a widely used measure for predictive accuracy for censored survival data:
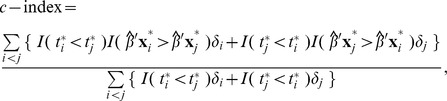



Larger 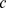-index corresponds to better prediction and 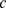-index = 0.5 means no prediction ability. The 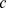-index is a less subjective measure than the LR-test and Cox-test; it requires no choice of a cut-off point for categorizing PI as in the LR-test, and requires no model-fitting as in the Cox-test. The 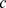-index is implemented in R (survConcordance routine in “survival” package) and other software [26].

### Simulation Set-up

The objective is to compare the prediction ability of the compound covariate method, the compound shrinkage method, and other existing methods. Comparative studies of Bovelstad et al. [17], van Wieringen et al. [18] and Bovelstad and Borgan [19] all demonstrated that ridge regression has the overall best predictive performance among many well-known survival prediction methods, including the univariate selection, forward selection, Lasso, principal components, supervised principal components, partial least squares, random forests, etc. On the other hand, Gui and Li [11], Segal [12] and Bovelstad and Borgan [19] still report some cases in which the Lasso-type methods perform better. Hence, we focus on the two benchmark methods of ridge regression and Lasso as representatives of existing methods.

We set the *p*-dimensional regression parameter 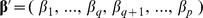 in the Cox model (1) with *p = *100. Note that we also considered *p = *50 and 200 but obtained similar results as reported in tables S1–1 ∼ S1–4 in Supporting Information S1. Consider a case, in which some of covariates are related to survival time; the coefficients of the first *q* covariates are nonzero and those of the remaining *p - q* covariates are zero. We examined (I) *sparse* cases (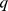 = 2, 4, 5 or 10) and (II) *less sparse* cases (*q* = 10, 15, 20 or 30). Note that both the sparse and non-sparse settings are plausible in biological problems [27]. For the covariates 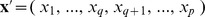, we adopt the following random effects models to introduce correlations among the covariates with a correlation coefficient equal to 0.5:

**Scenario 1 (tag genes):** Each of the *q* covariates is positively correlated to *s* covariates that have zero coefficients. Specifically, we set
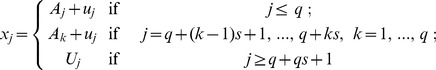



where 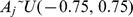, 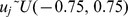, 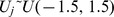, and they are independent of one another. This scenario represents the setting that *q* independent sets of genes are associated with survival; the (*s* +1) genes in each set are correlated, and after accounting for one “tag gene” in each set of genes, the other genes have no net effects on survival.

**Scenario 2 (gene pathway)**: The 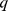 significant covariates are positively correlated. We set
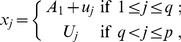
or



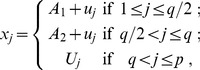



where 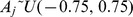, 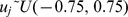, 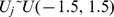, and they are independent of one another. The former represents the setting that there exists a “gene pathway” of *q* correlated genes that jointly affect survival, and the latter does for two gene pathways of *q*/2 correlated genes. Hence, scenario 2 represents a setting where the genes informative for survival are correlated while scenario 1 represents a setting where the informative genes are independent of each other.

For both scenarios, the covariates are standardized so that they have standard deviation 1. The Cox model in (1) with 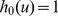 is chosen to generate survival times. Censoring times are generated from 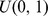, which yields moderate censoring (54∼63%). We first generate a training dataset of 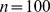 individuals, and calculate 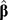, where 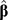 is the compound covariate, compound shrinkage, ridge regression or Lasso estimator. 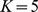 cross-validation is used to obtain the shrinkage parameters 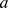 for the compound shrinkage estimator and 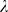 for ridge regression and Lasso estimators. Ridge regression and Lasso analyses are implemented through the R package “penalized” [21]. Then, we generate the test dataset of size 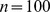, independently of the training dataset, to calculate the prediction measures of LR-test, Cox-test, Devi, and 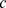-index.

In the subsequent simulations, we follow Bovelstad et al. [17] to compare the values from the LR-test, Cox-test, Devi and 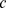-index by their median among 50 replications of training/test datasets.

### Simulation results

The results for the sparse cases (*q* = 2, 4, 5 or 10) are given in Table 1. The Lasso generally works best in all prediction measures. This pattern is only violated for the relatively large number of significant covariates (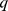 = 10) where the compound covariate or compound shrinkage method achieves better performance in terms of the LR-test, Cox-test and *c*-index. Ridge regression usually performs worst in terms of the LR-test, Cox-test, and 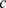-index. The compound shrinkage method is quite comparable in the LR-test, Cox-test, and 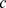-index to the compound covariate method in all cases.

**Table 1:** Simulation results under sparse cases with *p* = 100 and *n* = 100 based on 50 replications.

		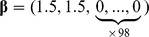 , *q* = 2				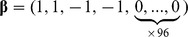 , *q* = 4			
		CC	CS	Ridge	Lasso	CC	CS	Ridge	Lasso
Scenario1, *s* = 4	LR-test	−5.89	−5.88	−4.99	−10.59	−4.71	−4.55	−4.75	−8.76
	Cox-test	−8.41	−8.26	−7.32	−13.80	−6.76	−7.06	−6.95	−11.73
	Devi	66.63	45.62	−29.48	−76.92	75.34	56.30	−25.75	−60.50
	*c*-index	0.772	0.768	0.752	0.859	0.750	0.751	0.750	0.825
	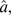 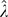	/	0.25	74.54	7.06	/	0.28	68.81	6.59
Scenario2	LR-test	−8.88	−9.35	−7.01	−12.39	−6.38	−6.74	−6.30	−11.40
	Cox-test	−12.16	−12.35	−9.64	−14.51	−9.27	−9.94	−8.77	−14.21
	Devi	−17.25	−26.02	−43.04	−95.39	−4.63	−11.32	−36.79	−84.14
	*c*-index	0.828	0.833	0.790	0.879	0.785	0.790	0.770	0.864
	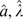	/	0.30	37.88	6.90	/	0.30	50.91	6.17
		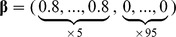 , *q* = 5				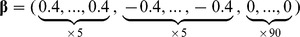 , *q* = 10			
		**CC**	**CS**	**Ridge**	**Lasso**	**CC**	**CS**	**Ridge**	**Lasso**
Scenario1, *s* = 4	LR-test	−3.88	−4.31	−4.21	−6.64	−2.28	−2.45	−2.40	−1.90
	Cox-test	−6.18	−6.19	−6.04	−9.47	−3.03	−3.03	−3.01	−2.86
	Devi	80.59	56.87	−21.44	−43.22	145.95	97.88	−9.28	−7.85
	*c*-index	0.725	0.722	0.722	0.790	0.659	0.656	0.652	0.649
	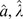	/	0.28	79.85	6.89	/	0.275	101.77	8.44
Scenario2	LR-test	−13.71	−13.69	−11.38	−14.52	−9.67	−9.34	−8.86	−9.65
	Cox-test	−15.18	−15.22	−14.04	−15.48	−12.68	−12.65	−11.34	−12.24
	Devi	−23.91	−34.13	−77.63	−107.14	8.563	−0.559	−55.62	−67.93
	*c*-index	0.886	0.885	0.862	0.889	0.843	0.835	0.822	0.838
	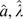	/	0.33	33.34	6.66	/	0.29	47.22	6.86

NOTE: For Scenario 1, each informative covariate is correlated with *s* non-informative covariates. For Scenario 2, the covariates for the right panel have two gene pathways and those for the left panel have one gene pathway. In each setting, *q* is the number of informative covariates (covariates with non-zero coefficients).

The four methods: **CC**  =  compound covariate, **CS**  =  compound shrinkage, **Ridge**  =  ridge regression, and **Lasso**  =  Lasso analyses are compared. The median values among the 50 replications for the LR-test (log_10_ P-value), Cox-test (log_10_ P-value), Devi, *c*-index, and tuning parameters 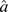 or 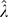 are reported.

The results for the less sparse cases (*q* = 10, 15, 20 or 30) are given in Table 2. Unlike the sparse cases, the Lasso usually performs worst in terms of the LR-test, Cox-test, and 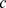-index, especially in scenario 1 where the Lasso estimates often result in the null model that has no prediction power (Devi = 0.000, *c*-index = 0.501∼ 0.538). Overall, the comparative performance of the compound covariate, compound shrinkage, and ridge regression methods are similar, but in scenario 2, the compound covariate and compound shrinkage methods perform better than the Lasso and ridge regression methods.

**Table 2:** Simulation results under less sparse cases with *p* = 100 and *n* = 100 based on 50 replications.

		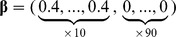 , *q* = 10				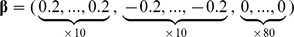 , *q* = 20			
		CC	CS	Ridge	Lasso	CC	CS	Ridge	Lasso
Scenario1, *s* = 2	LR-test	−1.99	−1.83	−1.88	−1.41	−1.22	−1.28	−1.29	−0.39
	Cox-test	−3.34	−3.34	−3.32	−2.22	−1.68	−1.69	−1.70	−0.45
	Devi	75.15	62.99	−10.09	−5.65	100.77	88.78	−3.79	0.000
	*c*-index	0.655	0.657	0.659	0.628	0.595	0.591	0.596	0.538
	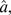 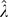	/	0.20	125.01	10.39	/	0.225	173.64	12.03
Scenario2	LR-test	−15.80	−14.84	−13.71	−14.80	−10.35	−9.49	−9.33	−9.11
	Cox-test	−15.35	−15.30	−15.05	−15.57	−13.23	−12.98	−12.30	−12.01
	Devi	59.54	48.07	−92.79	−103.80	114.48	75.17	−63.92	−60.30
	*c*-index	0.898	0.895	0.875	0.890	0.852	0.843	0.839	0.832
	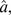 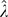	/	0.35	39.56	7.07	/	0.41	53.37	7.42
		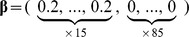 , *q* = 15				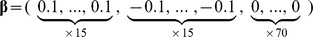 , *q* = 30			
		**CC**	**CS**	**Ridge**	**Lasso**	**CC**	**CS**	**Ridge**	**Lasso**
Scenario1, *s* = 2	LR-test	−1.10	−1.02	−0.95	−0.55	−0.55	−0.61	−0.61	−0.40
	Cox-test	−1.35	−1.27	−1.43	−0.42	−0.68	−0.66	−0.62	−0.22
	Devi	73.02	71.99	−1.20	0.000	96.21	89.26	−0.01	0.000
	*c*-index	0.601	0.598	0.605	0.529	0.552	0.548	0.559	0.501
	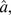 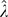	/	0.15	263.23	12.54	/	0.14	346.62	13.07
Scenario2	LR-test	−12.27	−11.84	−11.40	−11.41	−7.93	−6.80	−6.67	−6.05
	Cox-test	−12.87	−12.82	−12.77	−12.73	−10.55	−9.83	−9.65	−8.79
	Devi	291.82	177.76	−74.42	−71.46	326.63	141.46	−46.02	−38.22
	*c*-index	0.873	0.865	0.854	0.850	0.810	0.790	0.794	0.778
	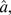 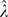	/	0.45	60.36	8.33	/	0.53	84.43	8.42

NOTE: For Scenario 1, each informative covariate is correlated with *s* non-informative covariates. For Scenario 2, the covariates for the right panel have two gene pathways and those for the left panel have one gene pathway. In each setting, *q* is the number of informative covariates (covariates with non-zero coefficients).

The four methods: **CC**  =  compound covariate, **CS**  =  compound shrinkage, **Ridge**  =  ridge regression, and **Lasso**  =  Lasso analyses are compared. The median values among the 50 replications for the LR-test (log_10_ P-value), Cox-test (log_10_ P-value), Devi, *c*-index, and tuning parameters 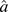 or 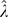 are reported.

In terms of the Devi, ridge regression and Lasso methods have much better performance than both the compound covariate and compound shrinkage methods. In fact, the Devi may be unfair to the proposed approach; the Devi measures a distance of 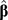 from the benchmark value of 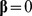, and the majority of regression coefficients obtained by ridge and Lasso are very close to or exactly 0 by construction. In contrast, the compound covariate and compound shrinkage methods have poorer performance in the Devi because they are not shrunk to 0. However, poorer performance in the Devi is not carried over to other measures based on association between the prognostic index and the survival time, i.e., the LR-test, Cox-test, and *c*-index.

To see the robustness of the proposed method to the cross-validation scheme, we perform the same set of simulations using 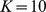 cross-validation in place of 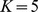. The results (not shown) are virtually identical to these in Tables 1 and 2. Hence, the performance of the compound shrinkage method is less affected by the number of folds used in the cross-validation.

Although we found no single best method across all cases, the comparative performance of the compound covariate and compound shrinkage methods with other methods is remarkable. Unlike ridge and Lasso analyses that may exhibit poor performance in certain specific cases, the compound covariate and compound shrinkage methods provide more stable performance across different settings with sparse/non-sparse, independent/correlated informative genes. This robustness property is desirable in practical applications.

We perform similar simulations by increasing the magnitude of non-zero coefficients. As reported in tables S1–5 and S1–6 in Supporting Information S1, prediction performance improved for all four methods, but the relative performances among them are similar to those seen in Tables 1 and 2.

### The Primary Biliary Cirrhosis Data Analysis

The primary biliary cirrhosis (PBC) data used in Tibshirani [10] contains 276 patients with 17 covariates. Among them, 111 patients died while others were censored. The covariates consist of a treatment indicator, age, sex, 5 categorical variables (ascites, hepatomegaly, spider, edema, and stage of disease) and 9 continuous variables (bilirubin, cholesterol, albumin, urine copper, alkarine, SGOT, triglycerides, platelet count, and prothrombine). We use log-transformed continuous covariates to get stable results. We compare the prediction performance over 50 random 2∶1 splits with 184 patients in the training set and 92 patients in the testing set.

Table 3 reports the results for comparing the compound covariate, compound shrinkage, multivariate Cox regression, ridge regression and Lasso analyses. Multivariate Cox regression analysis exhibits the worst performance, possibly due to a large number of covariates. The other four methods that adapt to high-dimensionality exhibit higher prediction power. Of these methods, the compound covariate method performs best in terms of the LR-test, Cox-test and *c*-index. This implies that the compound covariate has the highest ability to discriminate between the poor and good prognostic patients in the testing set. Notice that the poor Devi value of the compound covariate method does not affect its prediction power for patients’ prognosis.

**Table 3:** Performance of the five methods based on the primary biliary cirrhosis of the liver data.

	CC	CS	MultiCox	Ridge	Lasso
LR-test (log_10_ P-value)	−7.95	−7.00	−6.35	−6.98	−7.11
Cox-test (log_10_ P-value)	−12.49	−11.18	−10.71	−10.89	−10.71
*c*-index	0.846	0.829	0.825	0.843	0.834
Deviance	101.8	−39.9	−39.2	−49.4	−45.9
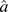 (CS), 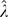 (Ridge/Lasso)	/	0.875	/	22.75	7.32

NOTE: The median among the 50 replications for the LR-test (log_10_ P-value), Cox-test (log_10_ P-value), Deviance, *c*-index, and tuning parameters 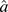 or 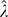 are reported. Smaller values of the LR-test, Cox-test and Deviance, and larger values of the *c*-index correspond to more accurate prediction performance.

The five methods: **CC**  =  compound covariate, **CS**  =  compound shrinkage, **MultiCox**  =  multivariate Cox regression, **Ridge**  =  ridge regression, and **Lasso**  =  Lasso analyses are compared.

**Table 4:** Performance of the five methods based on the non-small-cell lung cancer data of Chen et al. [6].

	97 genes				16 genes
	CC	CS	Ridge	Lasso	CC
LR-test (log_10_P-value)	−1.12	−0.75	−0.04	−0.15	−0.84*
Cox-test (log_10_P-value)	−0.19	−0.78	−0.03	−0.12	−0.16
*c*-index	0.581	0.606	0.535	0.544	0.584
Deviance	1520.3	68.4	15.2	15.8	439.5
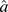 (CS), 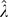 (Ridge/Lasso)	/	0.70	11.58	2.66	/
Computation time(sec)	0.41	895.9	2.12	3.05	0.06

NOTE: Smaller values of the LR-test (log_10_ P-value), Cox-test (log_10_ P-value) and Deviance, and larger values of the *c*-index correspond to more accurate prediction performance.

* If good and poor groups are separated by the median PI in the training set, the LR-test has P-value = 0.034 (log_10_ P-value = −1.47) with *n* = 28 in the good and *n* = 34 in the poor groups (the same result as Figure 1C of Chen et al. [6]).

The methods: **CC**  =  compound covariate (using 97 or 16 genes), **CS**  =  compound shrinkage, **Ridge**  =  ridge regression, and **Lasso**  =  Lasso analyses are compared.

### The Lung Cancer Data Analysis

The non-small-cell lung cancer data of Chen et al. [6] is available from http://www.ncbi.nlm.nih.gov/projects/geo/, with accession number GSE4882. The data contains 672 gene profiles for 125 lung cancer patients. Among them, 38 patients died while others were censored. We use a subset consisting of 485 genes whose coefficient of variation in expression values is greater than 3%. We divide the patients into 63∶62 training/test datasets as in Chen et al. [6]. Univariate Cox regression analysis based on the training set identifies 16 genes that are significantly related to survival (P-value <0.05). Chen et al. [6] used the 16 regression coefficients to classify the patients of the test dataset into good or poor status. This 16-gene method is a compound covariate analysis applied to the selected set of genes, though the compound covariate method is applicable for the full sets of 485 genes. To illustrate the compound covariate and the compound shrinkage methods with high-dimensional covariates, we select *p* = 97 genes whose P-values of the univariate analysis are less than 0.20 in the training dataset of *n* = 63, and set the coefficients of remaining genes to zero.

**Figure 2:**
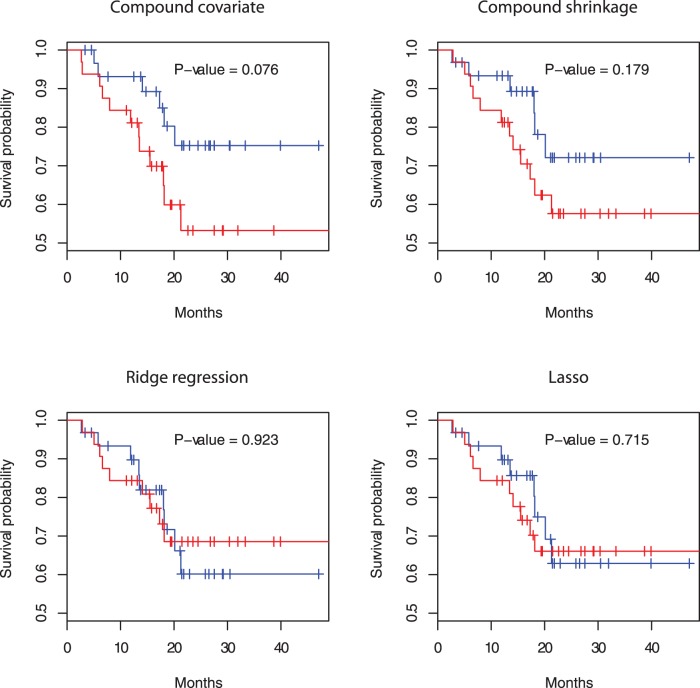
Kaplan-Meier curves for the 62 patients in the lung cancer data of Chen et al. [6]. Good (blue) and poor (red) groups are determined by the median of the PI’s in the test dataset.

**Figure 3:**
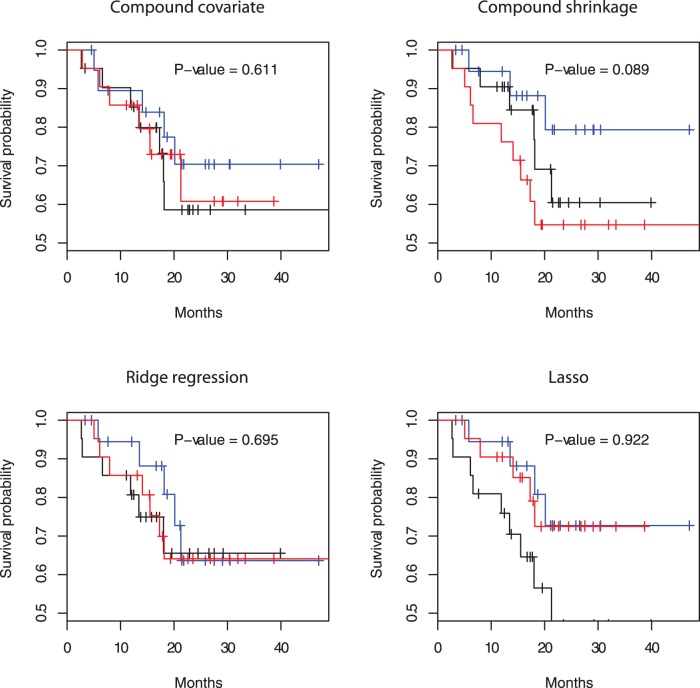
Kaplan-Meier curves for the 62 patients in the lung cancer data of Chen et al. [**6**]. Good (blue), medium (black), and poor (red) groups are determined by the tertile of the PI’s in the test dataset.

We compare the compound covariate, compound shrinkage, ridge regression, and Lasso methods as well as the 16-gene compound covariate method of Chen et al. [6]. The results are summarized in Table 4. In terms of the LR-test, the compound covariate method performs best, while, in terms of the Cox-test and 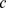-index, the compound shrinkage method performs best. Figure 2 shows that the two survival curves for the good and poor prognosis groups are best separated by the compound covariate method. However, Figure 3 shows that the Kaplan-Meier curves for the good, medium and poor prognosis groups cross one another and are less distinguishable by the compound covariate method. Here the good, medium, and poor groups are determined by the tertiles of the PI’s in the test datasets. On the other hand, the three Kaplan-Meier curves are well-distinguished in the compound shrinkage method, as implied by its best performance in the Cox-test and 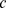-index (Figure 3; Table 4). This analysis suggests that, compared to the compound covariate method, the compound shrinkage method may provide more accurate ranking of patients’ risks with respect to their survival status. Although ridge regression and Lasso has much smaller deviance, it has poorer performance in the LR-test, Cox-test and 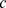-index.

**Figure 4:**
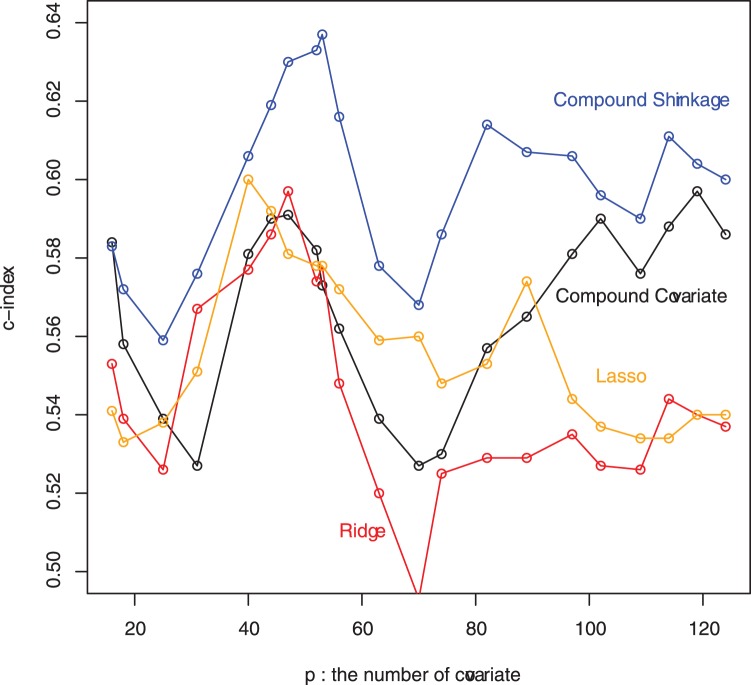
The *c*-index assessments of the four methods under varying number of top genes (*p* = 16 ∼ 124 ) in the lung cancer data of Chen et al. [6], where “top genes” refer to most strongly associated genes passing a univariate pre-filter for inclusion in the linear predictor (PI).

To see the robustness of the conclusion, comparison of the methods is made under various different numbers of genes, including 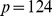 genes whose P-values of the univariate analysis are less than 0.25. As seen from the Supporting Information S2, the compound covariate method still performs best in terms of the LR-test. However, the compound shrinkage method still has the best performance in the Cox-test and 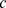-index, and it provides the best separation among the survival curves for the good, medium, and poor prognosis groups. In fact, the compound shrinkage method almost always has the best *c*-index values under varying number of genes passing a univariate pre-filter for inclusion in the PI (Figure 4). Hence, the conclusion is unchanged.

We also compared the computation time of the four methods in Table 4. The compound covariate method achieves the fastest computation time since it merely repeats *p* = 97 univariate Cox regressions using the R “coxph” routine. Ridge regression requires about 5 times and Lasso has about 7 times longer computation time than the compound covariate method. The compound shrinkage is decidedly the slowest, due to the cost of finding high-dimensional maxima 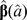 and 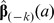.

## Analytical Results

### Large Sample Results for the Shrinkage Method

The first analytical result of the compound shrinkage method is the large sample consistency of the survival prediction. That is, as 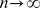 with fixed 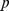, the estimated shrinkage parameter 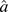 tends to 1 and the compound shrinkage estimator 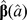 tends to the true parameter value 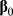. The second and more practically important result is a formula for the standard deviation of 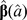 that may be useful for calculating P-values for each covariate.

To describe the analytical properties of 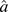 and 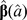, define, for 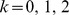,
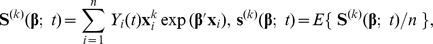



where 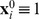, 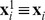, 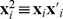 and 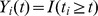 with 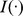 being an indicator function, and for 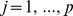,
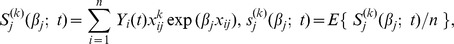


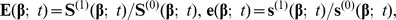


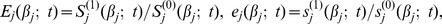


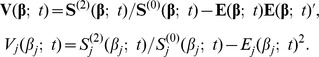



The score function defined as the derivative of 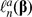 with respect to 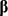 is given by
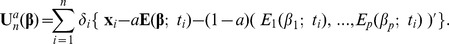



The observed Fisher information matrix, the negative of the Hessian of 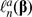, is
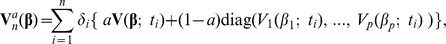



where 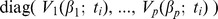 is the diagonal matrix with the diagonal element 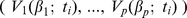. It is easy to verify that 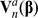 is positive semi-definite and hence 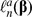 is concave for a given 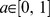. For 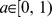, 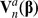 is typically positive definite and 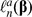 is strictly concave, which implies that 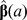 is unique even when 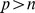.

Now we state the large sample results as 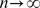 with fixed 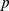; the proofs are given in Supporting Information S3. Assume that 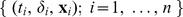 are independently and identically distributed under the model (1) with 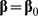, and 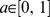 is a fixed constant. Applying martingale calculus and the concave property of 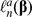 under mild regularity conditions (e.g. p.497–498 of [28]), we verify that 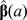 converges in probability to 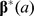, a solution to a 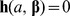 for a given 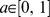 where
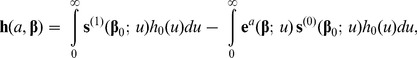
(8)


where 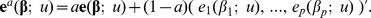


Note that, for 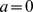, equation (8) is a multivariate generalization of equation (2–5) of Struthers and Kalbfleish [29] in the context of the misspecified Cox regression analysis. For 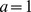, the solution to 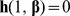 is 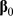, and hence 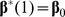.

*Proposition 1 (Consistency)*: As 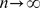, 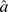 converges in probability to 1. Also, 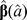 converges in probability to 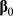.

*Proposition 2 (Asymptotic normality):* As 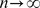, 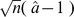 converges weakly to a mean zero normal distribution with variance 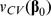. Also,
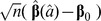
 converges weakly to a mean zero normal distribution with covariance matrix 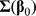. Explicit formulas for 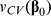 and 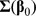 are derived in Supporting Information S3.

*Remark I.* We allow 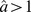 when 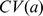 is maximized at 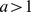.

*Remark II.* The asymptotic variance 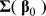 can be consistently estimated by 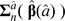, where
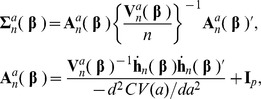


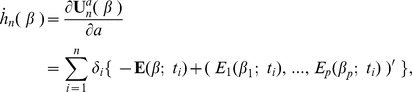



where 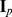 is the unit matrix of size 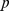. The estimator 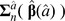 gives reasonable approximation to the variance of 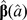 even when 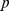 is large (see simulations for 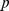 = 100 and 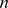 = 100 in Supporting Information S3). The variance estimate facilitates the Wald-type test for significance of the regression coefficients.

### Analytical Comparison with the Lasso and Ridge Regression

Unlike the Lasso and ridge regression in equations (3) and (4), which shrink the regression coefficients toward 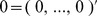, the compound shrinkage estimator is obtained by shrinking the coefficients toward the compound covariate estimator 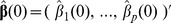.

We apply a statistical large sample theory on the misspecified Cox regression analysis [29], [30] to demonstrate that shrinking the regression coefficients toward the compound covariate estimator may be more informative than shrinking toward 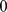 when covariates are independent. When 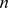 goes to infinity, the compound covariate estimator 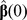 converges in probability to a vector 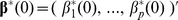, a solution to 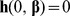 that is defined in equation (8). In general, 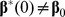, where 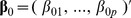 is the true parameter value in equation (1). Nevertheless, 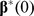 contains information about 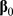. Without loss of generality, we will describe the properties of the first component 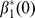 of 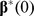, where the censoring is assumed independent of survival time and covariates.

(P1) If 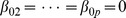, then 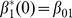.

(P2) Suppose that 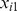 and 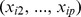 are independent for all 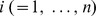. If 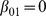, then 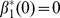. If 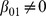, then 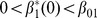 when 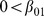, or 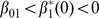 when 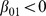.

The property (P1) is due to the fact that the univariate Cox estimate 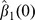 is obtained under the assumption that the hazard given 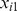 is of the form 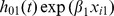, which is true when 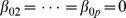 under equation (1). An important implication from the property (P1) is that, if 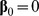, then 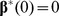 as well. The property (P2) is deduced from some known results of misspecified Cox regression analysis [29], [30]. The property (P2) implies that, if all the covariates are independent, the sign of each component of 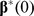 agrees with that of 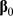, and 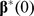 is closer to 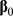 than 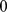. From the above properties, it is then expected that shrinking the regression coefficients toward 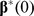 may be more informative than shrinking them toward 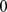. This gives an analytical reason justifying the proposed shrinkage method. The justification in the presence of correlations among covariates is analytically intractable, and hence is done by simulations and real data analysis as presented above.

The proposed shrinkage method has a natural interpretation under a setting of linear regression. Let 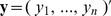 be the response vector and 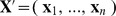 be the design matrix, where 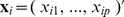 is the covariate for individual 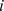. In the ordinary least square regression, we minimize the objective function 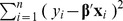. If 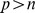, it does not have a unique minimizer since the design matrix 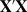 is singular. The proposed shrinkage scheme leads to minimizing.
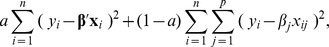



for some 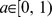. The minimizer of the above function is unique and written as
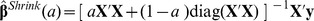



where 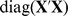 is a diagonal matrix with the same diagonal elements as in 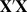. The singularity of 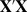 is thus resolved by reducing the off-diagonal values by a *multiplicative* factor 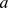. This is in contrast to ridge regression [13] where the diagonal values are increased by an *additive* factor 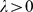, that is,
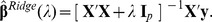



With complete shrinkage, the difference between the two estimators becomes evident since 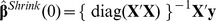 while 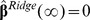.

### Computing Algorithms

Numerical maximization of 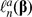 in equation (6) can be done through quasi-Newton type algorithms. For instance, the R “nlm” is a reliable routine to find the minimum of 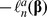 with a large 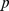.

Numerical maximization of 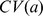 in equation (7) can be obtained by a grid search on finely selected values of 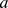 as commonly done in cross-validation [17], [24]. In our numerical studies we observe that the graph of 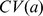 is always unimodal, and calculating 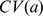 with smaller 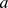 is always faster than with larger 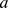. Utilizing these properties, we suggest the following computation algorithm, which is more efficient in computation than the “exhaustive search” procedure:

*Step 1:* Set 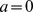 and a positive number 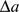 (e.g., 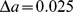), and calculate 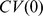.

*Step 2:* Set 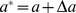. If 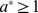, then go to Step 3. If 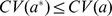, then go to Step 3. If 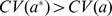, then set 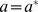 and return to Step 2.

*Step 3:* Stop the algorithm and set 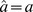.

### Conclusions

We have revisited a compound covariate prediction method for predicting survival outcomes with a large number of covariates. This method is popularly employed in medical studies, but its statistical performance has been less studied in the literature. We investigate the prediction power of the method by comparison with the well-known methods of ridge regression and Lasso, both of which adapt to a large number of covariates. The simulations demonstrate that the compound covariate method has better predictive power than ridge regression when only a few among a large number of covariates associate with the survival (i.e., sparse cases), and that it performs better than the Lasso when many of a large number of covariates simultaneously affect the survival (i.e., less sparse cases). The compound covariate method exhibits best predictive power among all the competitors in the primary biliary cirrhosis dataset, including the multivariate Cox regression, ridge regression and Lasso. In the even much higher dimensional lung cancer microarray data, where the multivariate Cox regression no longer applies, the compound covariate method similarly outperforms ridge regression and Lasso. Hence, the compound covariate method is a computationally attractive and powerful technique for survival prediction with a moderate or large number of covariates.

To further improve the prediction power of the compound covariate prediction, we propose a novel shrinkage type estimator for survival prediction with a large number of covariates. The new shrinkage scheme refines the compound covariate method by incorporating the multivariate likelihood information into the compound covariate predictor. Our simulation studies demonstrate that, in the sparse signal setting, the Lasso strongly outperforms the “non-sparse” methods, including ridge regression, compound covariate and compound shrinkage methods. On the other hand, in settings with less sparse signals, the compound covariate and compound shrinkage methods perform comparably to ridge regression, and all these methods outperform the Lasso method. Given that the non-sparse setting is not uncommon [27], and ridge regression shows best overall performance in several comparative prediction studies [17], [18], [19], the compound covariate and compound shrinkage methods have the potential to be useful alternatives. Our proposal also provides a novel framework of shrinkage estimation that encompasses the simple but effective compound covariate method as a special case. In the lung cancer data analysis we find that, the major advantage of the proposed compound shrinkage method over the compound covariate method is in its more accurate prediction of patient’s survival status. We also establish statistical large sample theories, including consistency and standard error estimation of the parameter estimator, for the proposed shrinkage method. Given these numerical and theoretical evidences, the proposed prediction scheme seems to be a method that can be reliably applied for survival prediction. The method is implemented by an R package “compound.Cox” available in CRAN at http://cran.r-project.org/.

A potential extension of the proposed shrinkage method is the development of covariate selection. This is clearly an important issue in microarrays in which the focus is to select genes that achieve good predictive power. If the gene selection is the main focus, we find the Lasso method offers an elegant solution since it gives an automatic way of selecting genes. In fact, the Lasso shows excellent performance when the signal is sparse, as shown in our simulation studies (Table 1). However, in the presence of a large number of informative genes (less sparse cases), the performance of the Lasso is less reliable since it tends to select only a few genes among them and often results in the null model with no prediction power (Table 2). A large number of informative genes are also encountered in the lymphoma data reported in Matsui [16], where the number of genes in the optimal set is 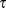 = 75 or 85. Matusi [16] suggests a *gene filtering* procedure that chooses the top 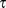 genes in terms of univariate Cox analyses, where 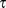 is the threshold that leads to the best predictive power in cross validation. Although this methodology is computationally simple, the top 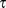 genes are based on univariate significance only. Hence, it is interesting to extend the gene filtering approach to take into account the combined, multivariate predictive information of genes using the proposed shrinkage method. We will leave this problem to a future research topic.

## Supporting Information

Supporting Information S1 **Simulation results for** ***p*** **= 50 and 200 (tables S1–1 ∼ S1–4) and for the increased magnitudes of the regression coefficients (tables S1–5, S1–6).**
(PDF)

Supporting Information S2 **Comparison of the prediction methods for the lung cancer data with** ***p*** **= 124 genes.**
(PDF)

Supporting Information S3 **Proofs of Propositions 1 and 2, variance estimation, and simulation results for variance estimation.**
(PDF)
