# Supplementary material for: Survival Prediction Based on Compound Covariate under Cox Proportional Hazard Models
Source: PLoS One. 2012 Oct 24;7(10):e47627. doi: 10.1371/journal.pone.0047627 (PMC3480451; doi:10.1371/journal.pone.0047627)
Supplement: Supporting Information S1 — Simulation results for p = 50 and 200 (tables S1–1 ∼ S1–4) and for the increased magnitudes of the regression coefficients (tables S1–5, S1–6). (PDF) [file pone.0047627.s001.pdf]

**Supporting Information S1:**

1) **Tables S1-1, S1-2, S1-3, S1-4: Simulation results for *p* = 50 and 200**

2) **Tables S1-5, S1-6: Results for the increased magnitudes of the regression coefficients**

**Table S1-1.** Simulation results under sparse cases with $p$ = 50 and $n$ = 100 based on 50 replications. The four methods: CC = compound covariate, CS = compound shrinkage, Ridge = ridge regression, and Lasso = Lasso analyses are compared. The median values among the 50 replications for the LR-test ($\log_{10}$ P-value), Cox-test ($\log_{10}$ P-value), Devi, $c$-index, and tuning parameters $\hat{a}$ or $\hat{\lambda}$ are reported.

| | | $\boldsymbol{\beta} = (1, 1, \underbrace{0, \ldots, 0}_{\times 48})$ ($q$=2) | | | | $\boldsymbol{\beta} = (0.5, 0.5, -0.5, -0.5, \underbrace{0, \ldots, 0}_{\times 46})$ ($q$=4) | | | |
|---|---|---|---|---|---|---|---|---|---|
| | | CC | CS | Ridge | Lasso | CC | CS | Ridge | Lasso |
| Scenario1 $s$ = 2 | LR-test | -3.95 | -3.95 | -3.87 | -6.41 | -2.26 | -2.35 | -2.01 | -2.97 |
| | Cox-test | -5.67 | -6.00 | -5.57 | -8.86 | -3.20 | -3.44 | -3.32 | -4.50 |
| | Devi | 8.95 | 4.50 | -19.45 | -36.50 | 32.82 | 24.52 | -10.78 | -15.95 |
| | $c$-index | 0.712 | 0.717 | 0.711 | 0.778 | 0.675 | 0.672 | 0.669 | 0.691 |
| | $\hat{a}$, $\hat{\lambda}$ | / | 0.23 | 51.99 | 7.17 | / | 0.225 | 70.79 | 7.05 |
| Scenario2 | LR-test | -7.31 | -7.60 | -7.01 | -8.63 | -3.87 | -3.84 | -3.54 | -4.13 |
| | Cox-test | -9.92 | -9.80 | -8.92 | -11.45 | -5.25 | -5.16 | -4.94 | -6.25 |
| | Devi | -28.82 | -30.55 | -37.68 | -59.05 | 9.48 | 8.23 | -17.75 | -28.07 |
| | $c$-index | 0.808 | 0.811 | 0.781 | 0.830 | 0.716 | 0.713 | 0.706 | 0.743 |
| | $\hat{a}$, $\hat{\lambda}$ | / | 0.23 | 35.19 | 7.36 | / | 0.19 | 45.46 | 7.17 |
| | | $\boldsymbol{\beta} = (\underbrace{0.4, \ldots, 0.4}_{\times 5}, \underbrace{0, \ldots, 0}_{\times 45})$ ($q$=5) | | | | $\boldsymbol{\beta} = (\underbrace{0.2, \ldots, 0.2}_{\times 5}, \underbrace{-0.2, \ldots, -0.2}_{\times 5}, \underbrace{0, \ldots, 0}_{\times 40})$ ($q$=10) | | | |
| | | CC | CS | Ridge | Lasso | CC | CS | Ridge | Lasso |
| Scenario1 $s$ = 2 | LR-test | -1.67 | -1.56 | -1.57 | -1.55 | -0.81 | -0.86 | -0.81 | -0.61 |
| | Cox-test | -2.49 | -2.38 | -2.43 | -2.68 | -1.15 | -1.06 | -1.07 | -0.43 |
| | Devi | 43.83 | 36.91 | -7.11 | -5.79 | 48.32 | 40.96 | -1.10 | 0.000 |
| | $c$-index | 0.628 | 0.625 | 0.621 | 0.640 | 0.590 | 0.578 | 0.587 | 0.544 |
| | $\hat{a}$, $\hat{\lambda}$ | / | 0.25 | 74.45 | 7.55 | / | 0.25 | 161.12 | 9.57 |
| Scenario2 | LR-test | -9.03 | -8.93 | -7.77 | -8.08 | -4.69 | -4.43 | -4.03 | -4.65 |
| | Cox-test | -10.78 | -10.74 | -9.65 | -10.26 | -6.41 | -6.38 | -5.93 | -5.76 |
| | Devi | 25.45 | 10.62 | -44.47 | -48.70 | 65.22 | 35.13 | -22.83 | -21.70 |
| | $c$-index | 0.811 | 0.802 | 0.794 | 0.806 | 0.738 | 0.731 | 0.723 | 0.717 |
| | $\hat{a}$, $\hat{\lambda}$ | / | 0.33 | 45.71 | 7.98 | / | 0.3 | 63.35 | 7.42 |

NOTE: For Scenario 1, each informative covariate is correlated with $s$ non-informative covariates. For Scenario 2, the covariates for the right panel have two gene pathways and those for the left panel have one gene pathway. In each setting, $q$ is the number of informative covariates (covariates with non-zero coefficients).

**Table S1-2.** Simulation results under less sparse cases with $p$ = 50 and $n$ = 100 based on 50 replications. The four methods: CC = compound covariate, CS = compound shrinkage, Ridge = ridge regression, and Lasso = Lasso analyses are compared. The median values among the 50 replications for the LR-test ($\log_{10}$ P-value), Cox-test ($\log_{10}$ P-value), Devi, $c$-index, and tuning parameters $\hat{a}$ or $\hat{\lambda}$ are reported.

| | | $\boldsymbol{\beta} = (\underbrace{0.2, ..., 0.2}_{\times 10}, \underbrace{0, ..., 0}_{\times 40})$ ($q$=10) | | | | $\boldsymbol{\beta} = (\underbrace{0.1, ..., 0.1}_{\times 10}, \underbrace{-0.1, ..., -0.1}_{\times 10}, \underbrace{0, ..., 0}_{\times 30})$ ($q$=20) | | | |
|---|---|---|---|---|---|---|---|---|---|
| | | CC | CS | Ridge | Lasso | CC | CS | Ridge | Lasso |
| Scenario1 | LR-test | -0.82 | -0.73 | -0.72 | -0.39 | -0.43 | -0.54 | -0.46 | -0.40 |
| $s$ = 0 | Cox-test | -0.84 | -0.84 | -0.84 | -0.39 | -0.65 | -0.73 | -0.73 | -0.23 |
| | Devi | 42.52 | 39.79 | -0.45 | 0.000 | 39.30 | 38.36 | -0.01 | 0.000 |
| | $c$-index | 0.563 | 0.562 | 0.563 | 0.504 | 0.556 | 0.557 | 0.549 | 0.513 |
| | $\hat{a}$, $\hat{\lambda}$ | / | 0.13 | 178.30 | 12.30 | / | 0.13 | 285.41 | 11.62 |
| Scenario2 | LR-test | -8.48 | -7.93 | -7.82 | -7.41 | -4.48 | -4.07 | -3.94 | -2.96 |
| | Cox-test | -9.85 | -9.44 | -9.53 | -9.43 | -6.23 | -5.78 | -5.69 | -4.70 |
| | Devi | 179.46 | 41.91 | -45.07 | -43.79 | 178.38 | 36.91 | -22.50 | -17.15 |
| | $c$-index | 0.814 | 0.796 | 0.797 | 0.792 | 0.735 | 0.716 | 0.717 | 0.699 |
| | $\hat{a}$, $\hat{\lambda}$ | / | 0.55 | 58.73 | 8.23 | / | 0.55 | 76.26 | 7.89 |
| | | $\boldsymbol{\beta} = (\underbrace{0.1, ..., 0.1}_{\times 15}, \underbrace{0, ..., 0}_{\times 35})$ ($q$=15) | | | | $\boldsymbol{\beta} = (\underbrace{0.05, ..., 0.05}_{\times 15}, \underbrace{-0.05, ..., -0.05}_{\times 15}, \underbrace{0, ..., 0}_{\times 20})$ ($q$=30) | | | |
| | | CC | CS | Ridge | Lasso | CC | CS | Ridge | Lasso |
| Scenario1 | LR-test | -0.31 | -0.26 | -0.45 | -0.41 | -0.29 | -0.27 | -0.25 | -0.27 |
| $s$ = 0 | Cox-test | -0.35 | -0.38 | -0.38 | -0.18 | -0.35 | -0.37 | -0.35 | -0.02 |
| | Devi | 43.88 | 41.63 | 0.000 | 0.000 | 44.48 | 44.86 | 0.002 | 0.000 |
| | $c$-index | 0.512 | 0.510 | 0.513 | 0.500 | 0.509 | 0.511 | 0.512 | 0.500 |
| | $\hat{a}$, $\hat{\lambda}$ | / | 0.11 | 456.08 | 13.10 | / | 0.125 | 487.07 | 12.69 |
| Scenario2 | LR-test | -5.55 | -4.77 | -5.75 | -4.57 | -2.77 | -2.39 | -2.63 | -1.73 |
| | Cox-test | -7.28 | -6.20 | -6.63 | -6.01 | -4.19 | -3.35 | -3.96 | -2.56 |
| | Devi | 361.45 | 62.38 | -29.24 | -25.38 | 325.48 | 54.74 | -13.37 | -8.56 |
| | $c$-index | 0.761 | 0.736 | 0.751 | 0.735 | 0.680 | 0.663 | 0.679 | 0.643 |
| | $\hat{a}$, $\hat{\lambda}$ | / | 0.63 | 75.68 | 8.41 | / | 0.575 | 115.11 | 8.06 |

NOTE: For Scenario 1, each informative covariate is correlated with $s$ non-informative covariates. For Scenario 2, the covariates for the right panel have two gene pathways and those for the left panel have one gene pathway. In each setting, $q$ is the number of informative covariates (covariates with non-zero coefficients).

**Table S1-3.** Simulation results under sparse cases with $p$ = 200 and $n$ = 100 based on 50 replications. The four methods: CC = compound covariate, CS = compound shrinkage, Ridge = ridge regression, and Lasso = Lasso analyses are compared. The median values among the 50 replications for the LR-test ($\log_{10}$ P-value), Cox-test ($\log_{10}$ P-value), Devi, $c$-index, and tuning parameters $\hat{a}$ or $\hat{\lambda}$ are reported.

| | | $\boldsymbol{\beta}=(2,2,\underbrace{0,\ldots,0}_{\times 198})$ ($q$=2) | | | | $\boldsymbol{\beta}=(1.5,1.5,-1.5,-1.5,\underbrace{0,\ldots,0}_{\times 196})$ ($q$=4) | | | |
|---|---|---|---|---|---|---|---|---|---|
| | | CC | CS | Ridge | Lasso | CC | CS | Ridge | Lasso |
| Scenario1 | LR-test | -6.59 | -5.99 | -5.51 | -12.50 | -5.66 | -5.57 | -5.38 | -12.06 |
| $s$ = 8 | Cox-test | -8.14 | -8.46 | -7.69 | -15.48 | -7.86 | -7.87 | -7.93 | -15.65 |
| | Devi | 208.12 | 137.72 | -31.66 | -97.74 | 211.17 | 136.61 | -30.44 | -94.04 |
| | $c$-index | 0.777 | 0.778 | 0.755 | 0.879 | 0.755 | 0.762 | 0.753 | 0.879 |
| | $\hat{a}$, $\hat{\lambda}$ | / | 0.3 | 124.55 | 7.35 | / | 0.33 | 115.57 | 6.31 |
| Scenario2 | LR-test | -9.06 | -9.69 | -6.36 | -14.88 | -6.08 | -6.62 | -5.49 | -15.00 |
| | Cox-test | -12.54 | -12.94 | -8.68 | -16.69 | -8.56 | -9.52 | -8.26 | -19.91 |
| | Devi | 17.92 | 12.53 | -34.41 | -121.12 | 45.02 | 22.54 | -32.75 | -121.32 |
| | $c$-index | 0.827 | 0.836 | 0.769 | 0.908 | 0.773 | 0.778 | 0.760 | 0.913 |
| | $\hat{a}$, $\hat{\lambda}$ | / | 0.31 | 82.56 | 6.91 | / | 0.28 | 93.58 | 6.07 |
| | | $\boldsymbol{\beta}=(\underbrace{1,\ldots,1}_{\times 5},\underbrace{0,\ldots,0}_{\times 195})$ ($q$=5) | | | | $\boldsymbol{\beta}=(\underbrace{0.8,\ldots,0.8}_{\times 5},\underbrace{-0.8,\ldots,-0.8}_{\times 5},\underbrace{0,\ldots,0}_{\times 190})$ ($q$=10) | | | |
| | | CC | CS | Ridge | Lasso | CC | CS | Ridge | Lasso |
| Scenario1 | LR-test | -5.72 | -5.30 | -4.85 | -7.98 | -5.55 | -5.45 | -5.34 | -6.73 |
| $s$ = 8 | Cox-test | -7.50 | -7.37 | -7.23 | -11.87 | -7.53 | -7.54 | -7.82 | -9.36 |
| | Devi | 294.76 | 218.91 | -25.07 | -58.21 | 311.81 | 204.79 | -29.38 | -40.73 |
| | $c$-index | 0.754 | 0.749 | 0.746 | 0.829 | 0.739 | 0.750 | 0.741 | 0.775 |
| | $\hat{a}$, $\hat{\lambda}$ | / | 0.29 | 130.63 | 7.22 | / | 0.33 | 127.06 | 7.54 |
| Scenario2 | LR-test | -14.66 | -14.26 | -11.90 | -17.56 | -12.64 | -12.74 | -11.68 | -15.56 |
| | Cox-test | -15.65 | -15.65 | -14.74 | -20.22 | -14.98 | -15.26 | -14.27 | -18.23 |
| | Devi | -2.320 | -7.88 | -73.28 | -129.09 | -10.19 | -23.95 | -72.81 | -127.08 |
| | $c$-index | 0.889 | 0.889 | 0.857 | 0.918 | 0.865 | 0.868 | 0.855 | 0.912 |
| | $\hat{a}$, $\hat{\lambda}$ | / | 0.39 | 55.92 | 6.55 | / | 0.33 | 54.63 | 6.01 |

NOTE: For Scenario 1, each informative covariate is correlated with $s$ non-informative covariates. For Scenario 2, the covariates for the right panel have two gene pathways and those for the left panel have one gene pathway. In each setting, $q$ is the number of informative covariates (covariates with non-zero coefficients).

**Table S1-4.** Simulation results under less sparse cases with $p$ = 200 and $n$ = 100 based on 50 replications. The four methods: CC = compound covariate, CS = compound shrinkage, Ridge = ridge regression, and Lasso = Lasso analyses are compared. The median values among the 50 replications for the LR-test ($\log_{10}$ P-value), Cox-test ($\log_{10}$ P-value), Devi, $c$-index, and tuning parameters $\hat{a}$ or $\hat{\lambda}$ are reported.

| | | $\boldsymbol{\beta} = (\underbrace{0.8,\ldots,0.8}_{\times 10}, \underbrace{0,\ldots,0}_{\times 40})$ ($q$=10) | | | | $\boldsymbol{\beta} = (\underbrace{0.4,\ldots,0.4}_{\times 10}, \underbrace{-0.4,\ldots,-0.4}_{\times 10}, \underbrace{0,\ldots,0}_{\times 180})$ ($q$=20) | | | |
|---|---|---|---|---|---|---|---|---|---|
| | | CC | CS | Ridge | Lasso | CC | CS | Ridge | Lasso |
| Scenario1 | LR-test | -4.26 | -4.44 | -4.79 | -6.21 | -3.07 | -3.51 | -3.19 | -1.00 |
| $s$ = 4 | Cox-test | -7.27 | -7.34 | -7.11 | -9.50 | -4.44 | -4.40 | -4.44 | -1.72 |
| | Devi | 131.98 | 113.33 | -25.10 | -41.82 | 199.58 | 153.35 | -14.61 | -3.11 |
| | $c$-index | 0.740 | 0.741 | 0.734 | 0.781 | 0.686 | 0.685 | 0.689 | 0.605 |
| | $\hat{a}$, $\hat{\lambda}$ | / | 0.25 | 128.65 | 7.77 | / | 0.25 | 186.38 | 11.82 |
| Scenario2 | LR-test | -Inf | -Inf | -Inf | -Inf | -15.57 | -15.65 | -14.19 | -15.16 |
| | Cox-test | -Inf | -Inf | -Inf | -Inf | -Inf | -Inf | -Inf | -Inf |
| | Devi | -48.67 | -46.45 | -125.89 | -167.76 | -0.26 | 2.82 | -101.29 | -114.36 |
| | $c$-index | 0.936 | 0.936 | 0.913 | 0.944 | 0.899 | 0.901 | 0.887 | 0.898 |
| | $\hat{a}$, $\hat{\lambda}$ | / | 0.45 | 38.46 | 5.76 | / | 0.43 | 46.06 | 6.56 |
| | | $\boldsymbol{\beta} = (\underbrace{0.4,\ldots,0.4}_{\times 15}, \underbrace{0,\ldots,0}_{\times 185})$ ($q$=15) | | | | $\boldsymbol{\beta} = (\underbrace{0.2,\ldots,0.2}_{\times 15}, \underbrace{-0.2,\ldots,-0.2}_{\times 15}, \underbrace{0,\ldots,0}_{\times 170})$ ($q$=30) | | | |
| | | CC | CS | Ridge | Lasso | CC | CS | Ridge | Lasso |
| Scenario1 | LR-test | -2.48 | -2.31 | -2.26 | -1.21 | -1.13 | -1.16 | -1.27 | -0.51 |
| $s$ = 2 | Cox-test | -3.57 | -3.53 | -3.68 | -1.80 | -1.54 | -1.46 | -1.74 | -0.63 |
| | Devi | 147.39 | 126.59 | -11.43 | -1.04 | 163.78 | 153.78 | -2.72 | 0.064 |
| | $c$-index | 0.668 | 0.664 | 0.669 | 0.611 | 0.595 | 0.603 | 0.609 | 0.551 |
| | $\hat{a}$, $\hat{\lambda}$ | / | 0.25 | 171.12 | 10.27 | / | 0.225 | 329.95 | 12.24 |
| Scenario2 | LR-test | -Inf | -Inf | -Inf | -Inf | -13.56 | -13.05 | -12.27 | -12.05 |
| | Cox-test | -Inf | -Inf | -Inf | -Inf | -15.23 | -15.41 | -15.11 | -14.86 |
| | Devi | 54.49 | 33.15 | -121.91 | -131.84 | 157.47 | 108.52 | -91.29 | -88.23 |
| | $c$-index | 0.929 | 0.929 | 0.913 | 0.916 | 0.894 | 0.891 | 0.877 | 0.875 |
| | $\hat{a}$, $\hat{\lambda}$ | / | 0.45 | 48.34 | 6.95 | / | 0.4 | 62.29 | 7.24 |

NOTE: For Scenario 1, each informative covariate is correlated with $s$ non-informative covariates. For Scenario 2, the covariates for the right panel have two gene pathways and those for the left panel have one gene pathway. In each setting, $q$ is the number of informative covariates (covariates with non-zero coefficients).

**Table S1-5.** Simulation results under sparse cases with $p$ = 100 and $n$ = 100 based on 50 replications. The four methods: CC = compound covariate, CS = compound shrinkage, Ridge = ridge regression, and Lasso = Lasso analyses are compared. The median values among the 50 replications for the LR-test ($\log_{10}$ P-value), Cox-test ($\log_{10}$ P-value), Devi, $c$-index, and tuning parameters $\hat{a}$ or $\hat{\lambda}$ are reported.

| | | $\boldsymbol{\beta} = (2, 2, \underbrace{0, ..., 0}_{\times 98})$ ($q$=2) | | | | $\boldsymbol{\beta} = (1.5, 1.5, -1.5, -1.5, \underbrace{0, ..., 0}_{\times 96})$ ($q$=4) | | | |
|---|---|---|---|---|---|---|---|---|---|
| | | CC | CS | Ridge | Lasso | CC | CS | Ridge | Lasso |
| Scenario1 | LR-test | -6.88 | -7.72 | -6.74 | -13.35 | -6.74 | -7.37 | -6.98 | -12.28 |
| $s$ = 4 | Cox-test | -9.98 | -10.52 | -9.59 | -15.48 | -9.09 | -9.55 | -9.67 | -15.26 |
| | Devi | 49.32 | 27.99 | -39.25 | -101.35 | 42.94 | 13.25 | -41.07 | -96.77 |
| | $c$-index | 0.800 | 0.800 | 0.780 | 0.888 | 0.779 | 0.786 | 0.790 | 0.889 |
| | $\hat{a}$, $\hat{\lambda}$ | / | 0.28 | 59.09 | 6.67 | / | 0.30 | 54.54 | 5.94 |
| Scenario2 | LR-test | -11.68 | -11.94 | -8.98 | -15.26 | -8.70 | -9.39 | -8.82 | -15.57 |
| | Cox-test | -11.05 | -14.86 | -12.28 | -Inf | -12.33 | -13.12 | -12.07 | -Inf |
| | Devi | -53.56 | -58.90 | -58.14 | -124.64 | -29.05 | -46.65 | -57.25 | -123.19 |
| | $c$-index | 0.857 | 0.866 | 0.825 | 0.909 | 0.819 | 0.834 | 0.822 | 0.907 |
| | $\hat{a}$, $\hat{\lambda}$ | / | 0.33 | 32.29 | 5.87 | / | 0.38 | 32.59 | 5.42 |

| | | $\boldsymbol{\beta} = (\underbrace{1, ..., 1}_{\times 5}, \underbrace{0, ..., 0}_{\times 95})$ ($q$=5) | | | | $\boldsymbol{\beta} = (\underbrace{0.8, ..., 0.8}_{\times 5}, \underbrace{-0.8, ..., -0.8}_{\times 5}, \underbrace{0, ..., 0}_{\times 90})$ ($q$=10) | | | |
|---|---|---|---|---|---|---|---|---|---|
| | | CC | CS | Ridge | Lasso | CC | CS | Ridge | Lasso |
| Scenario1 | LR-test | -4.94 | -5.27 | -5.29 | -8.88 | -5.08 | -5.46 | -5.24 | -7.56 |
| $s$ = 4 | Cox-test | -7.57 | -7.80 | -7.87 | -12.23 | -7.20 | -7.80 | -7.76 | -10.61 |
| | Devi | 66.77 | 42.04 | -31.02 | -61.66 | 112.96 | 47.51 | -32.24 | -51.94 |
| | $c$-index | 0.751 | 0.755 | 0.754 | 0.834 | 0.749 | 0.762 | 0.756 | 0.803 |
| | $\hat{a}$, $\hat{\lambda}$ | / | 0.3 | 62.34 | 6.39 | / | 0.35 | 53.89 | 6.10 |
| Scenario2 | LR-test | -15.41 | -15.26 | -12.86 | -Inf | -14.72 | -14.46 | -13.77 | -15.65 |
| | Cox-test | -Inf | -Inf | -15.50 | -Inf | -Inf | -Inf | -15.95 | -Inf |
| | Devi | -53.41 | -64.37 | -95.14 | -129.73 | -92.39 | -96.40 | -101.63 | -135.72 |
| | $c$-index | 0.905 | 0.909 | 0.884 | 0.917 | 0.902 | 0.905 | 0.890 | 0.923 |
| | $\hat{a}$, $\hat{\lambda}$ | / | 0.35 | 28.06 | 5.93 | / | 0.39 | 22.78 | 5.07 |

NOTE: For Scenario 1, each informative covariate is correlated with $s$ non-informative covariates. For Scenario 2, the covariates for the right panel have two gene pathways and those for the left panel have one gene pathway. In each setting, $q$ is the number of informative covariates (covariates with non-zero coefficients).

**Table S1-6.** Simulation results under less sparse cases with $p$ = 100 and $n$ = 100 based on 50 replications. The four methods: CC = compound covariate, CS = compound shrinkage, Ridge = ridge regression, and Lasso = Lasso analyses are compared. The median values among the 50 replications for the LR-test ($\log_{10}$ P-value), Cox-test ($\log_{10}$ P-value), Devi, $c$-index, and tuning parameters $\hat{a}$ or $\hat{\lambda}$ are reported.

| | | $\boldsymbol{\beta} = (\underbrace{0.8,\ldots,0.8}_{\times 10}, \underbrace{0,\ldots,0}_{\times 90})$ ($q$=10) | | | | $\boldsymbol{\beta} = (\underbrace{0.4,\ldots,0.4}_{\times 10}, \underbrace{-0.4,\ldots,-0.4}_{\times 10}, \underbrace{0,\ldots,0}_{\times 80})$ ($q$=20) | | | |
|---|---|---|---|---|---|---|---|---|---|
| | | CC | CS | Ridge | Lasso | CC | CS | Ridge | Lasso |
| Scenario1 | LR-test | -4.98 | -4.89 | -5.07 | -7.52 | -3.49 | -3.47 | -3.42 | -1.57 |
| $s$ = 2 | Cox-test | -6.95 | -7.50 | -7.79 | -11.55 | -4.82 | -5.24 | -4.96 | -2.45 |
| | Devi | 42.37 | 24.75 | -32.39 | -50.79 | 72.30 | 45.07 | -20.02 | -6.08 |
| | $c$-index | 0.738 | 0.748 | 0.752 | 0.810 | 0.693 | 0.700 | 0.703 | 0.644 |
| | $\hat{a}$, $\hat{\lambda}$ | / | 0.29 | 64.11 | 6.37 | / | 0.29 | 73.33 | 8.59 |
| Scenario2 | LR-test | -Inf | -Inf | -Inf | -Inf | -15.65 | -15.95 | -14.58 | -15.11 |
| | Cox-test | -Inf | -Inf | -Inf | -Inf | -Inf | -Inf | -Inf | -Inf |
| | Devi | -115.29 | -107.94 | -144.96 | -172.36 | -50.66 | -55.42 | -117.07 | -121.55 |
| | $c$-index | 0.945 | 0.944 | 0.928 | 0.943 | 0.918 | 0.919 | 0.906 | 0.913 |
| | $\hat{a}$, $\hat{\lambda}$ | / | 0.40 | 22.40 | 5.11 | / | 0.40 | 28.38 | 5.61 |

| | | $\boldsymbol{\beta} = (\underbrace{0.4,\ldots,0.4}_{\times 15}, \underbrace{0,\ldots,0}_{\times 85})$ ($q$=15) | | | | $\boldsymbol{\beta} = (\underbrace{0.2,\ldots,0.2}_{\times 15}, \underbrace{-0.2,\ldots,-0.2}_{\times 15}, \underbrace{0,\ldots,0}_{\times 70})$ ($q$=30) | | | |
|---|---|---|---|---|---|---|---|---|---|
| | | CC | CS | Ridge | Lasso | CC | CS | Ridge | Lasso |
| Scenario1 | LR-test | -3.20 | -2.93 | -2.92 | -1.84 | -1.56 | -1.61 | -1.60 | -0.44 |
| $s$ = 2 | Cox-test | -3.99 | -4.28 | -4.31 | -2.72 | -1.92 | -1.89 | -2.09 | -0.31 |
| | Devi | 46.00 | 41.62 | -14.22 | -4.91 | 80.68 | 75.95 | -5.02 | 0.000 |
| | $c$-index | 0.690 | 0.693 | 0.693 | 0.642 | 0.629 | 0.627 | 0.629 | 0.536 |
| | $\hat{a}$, $\hat{\lambda}$ | / | 0.20 | 89.77 | 8.81 | / | 0.20 | 140.66 | 11.89 |
| Scenario2 | LR-test | -Inf | -Inf | -Inf | -Inf | -13.82 | -13.61 | -13.21 | -12.08 |
| | Cox-test | -Inf | -Inf | -Inf | -Inf | -15.48 | -15.48 | -15.41 | -14.86 |
| | Devi | 4.36 | 21.26 | -133.94 | -136.69 | 108.27 | 63.39 | -98.83 | -90.90 |
| | $c$-index | 0.935 | 0.936 | 0.922 | 0.922 | 0.903 | 0.901 | 0.890 | 0.876 |
| | $\hat{a}$, $\hat{\lambda}$ | / | 0.43 | 32.73 | 5.88 | / | 0.40 | 45.55 | 7.06 |

NOTE: For Scenario 1, each informative covariate is correlated with $s$ non-informative covariates. For Scenario 2, the covariates for the right panel have two gene pathways and those for the left panel have one gene pathway. In each setting, $q$ is the number of informative covariates (covariates with non-zero coefficients).
