# Supplementary material for: Survival Prediction Based on Compound Covariate under Cox Proportional Hazard Models
Source: PLoS One. 2012 Oct 24;7(10):e47627. doi: 10.1371/journal.pone.0047627 (PMC3480451; doi:10.1371/journal.pone.0047627)
Supplement: Supporting Information S2 — Comparison of the prediction methods for the lung cancer data with p = 124 genes. (PDF) [file pone.0047627.s002.pdf]

Supporting Information S2:

Comparison of the four prediction methods for the lung cancer data with *p* = 124 genes.

Table S2. Performance of the four methods based on the non-small-cell lung cancer data of Chen et al. (2007) with *p* = 124 genes

	Compound covariate	Compound shrinkage	Ridge regression	Lasso
LR-test ($\log_{10}$ P-value)	-1.07	-0.52	-0.04	-0.15
Cox-test ($\log_{10}$ P-value)	-0.16	-0.94	-0.10	-0.13
c-index	0.586	0.600	0.537	0.540
Deviance	1781.2	68.3	12.7	13.9
$\hat{a}$, $\hat{\lambda}$	/	0.7	12.39	2.91

NOTE: Smaller values of the LR-test, Cox-test and Deviance, and larger values of the *c*-index correspond to accurate prediction performance.

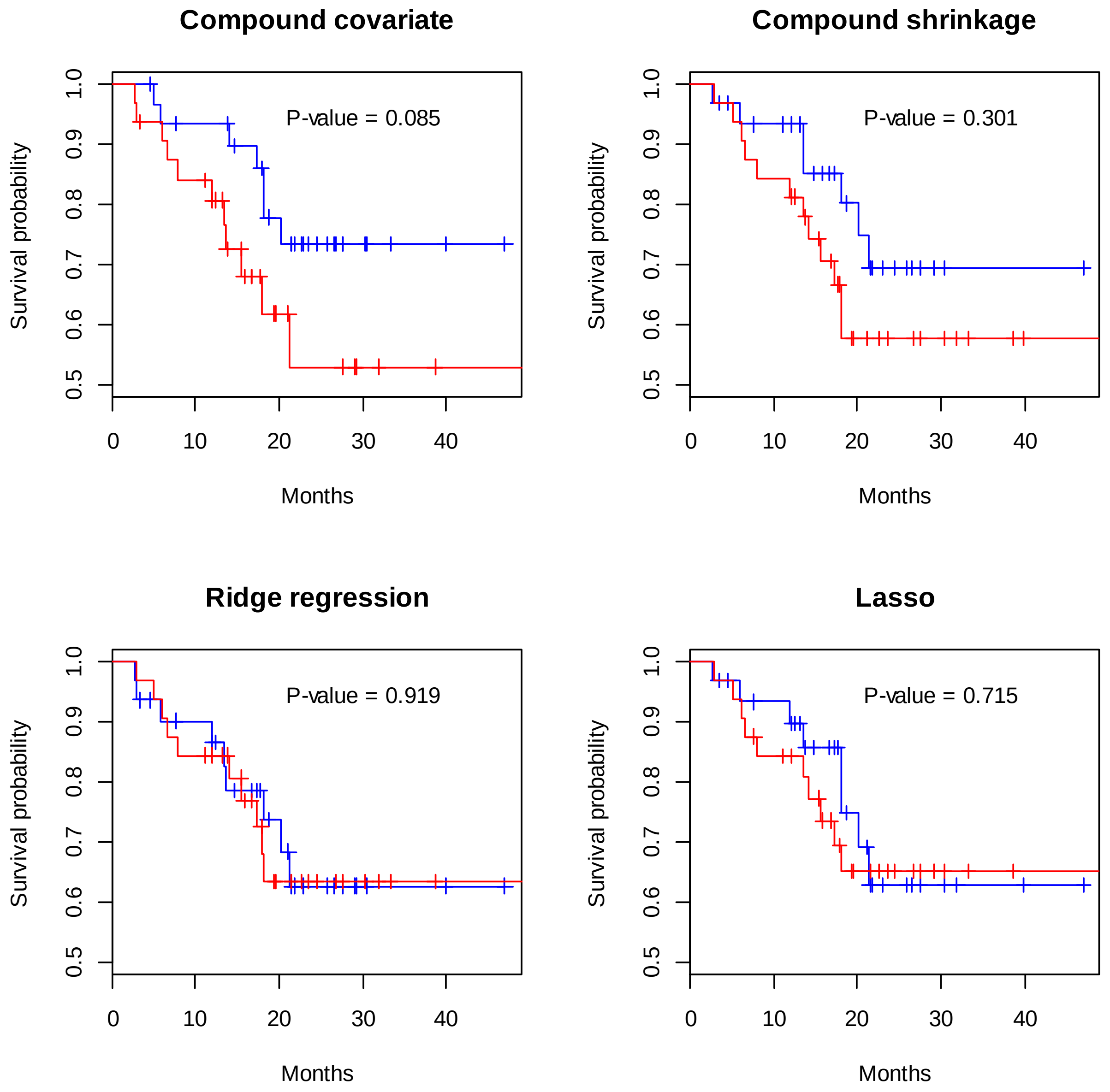

Figure S2-a. Kaplan-Meier curves for the 62 patients in the lung cancer data of Chen et al. (2007) with p = 124 genes. Good (blue line) and poor (red line) groups are determined by the median of the PI's in the test dataset.

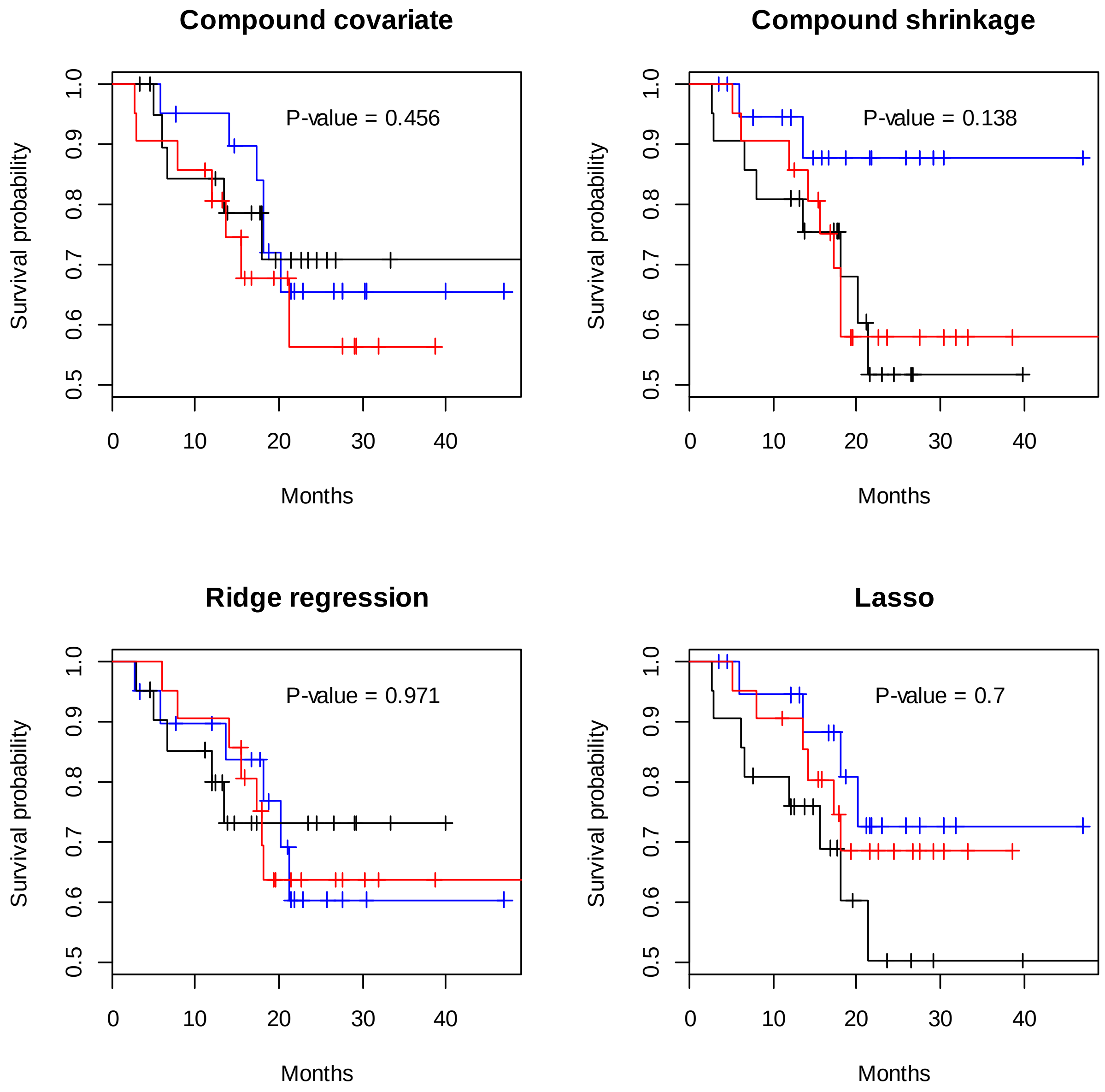

Figure S2-b. Kaplan-Meier curves for the 62 patients in the lung cancer data of Chen et al. (2007) with p = 124 genes. Good (blue line), medium (black line), and poor (red line) groups are determined by the tertile of the PI's in the test dataset.
