# Supplementary material for: Survival Prediction Based on Compound Covariate under Cox Proportional Hazard Models
Source: PLoS One. 2012 Oct 24;7(10):e47627. doi: 10.1371/journal.pone.0047627 (PMC3480451; doi:10.1371/journal.pone.0047627)
Supplement: Supporting Information S3 — Proofs of Propositions 1 and 2, variance estimation, and simulation results for variance estimation. (PDF) [file pone.0047627.s003.pdf]

Supporting Information S3

- **Proofs of Propositions 1 and 2**
- **Variance estimation**
- **Simulation results for variance estimation**

Proofs of Propositions 1 and 2:

The asymptotic results are stated under $n \to \infty$ with a fixed p. Let

$$\mathbf{v}(\boldsymbol{\beta};t) = \mathbf{s}^{(2)}(\boldsymbol{\beta};t)/\mathbf{s}^{(0)}(\boldsymbol{\beta};t) - \mathbf{e}(\boldsymbol{\beta};t)\mathbf{e}(\boldsymbol{\beta};t)', \quad v_j(\beta_j;t) = s_j^{(2)}(\beta_j;t)/s_j^{(0)}(\beta_j;t) - e_j(\beta_j;t)^2,$$

and

$$\mathbf{v}^a(\boldsymbol{\beta}) = \int_0^\infty [a\mathbf{v}(\boldsymbol{\beta};u) + (1-a)\mathrm{diag}\{v_1(\beta_1;u), ..., v_p(\beta_p;u)\}]\, \mathbf{s}^{(0)}(\boldsymbol{\beta}_0;u)h_0(u)du.$$

To prove propositions 1 and 2, we need the following lemmas:

Lemma 1 (asymptotic linear expression): For arbitrary $\boldsymbol{\beta}$,

$$\frac{1}{\sqrt{n}}\mathbf{U}_n^a(\boldsymbol{\beta}) = \frac{1}{\sqrt{n}}\sum_{i=1}^n \mathbf{w}_i^a(\boldsymbol{\beta}) + o_P(1),$$

where

$$\mathbf{w}_i^a(\boldsymbol{\beta}) = \int_0^\infty \{\mathbf{x}_i - \mathbf{e}^a(\boldsymbol{\beta};u)\}dN_i(u) - \int_0^\infty Y_i(u)\left\{ a\frac{\mathbf{x}_i e^{\boldsymbol{\beta}'\mathbf{x}_i} - \mathbf{e}(\boldsymbol{\beta};u)e^{\boldsymbol{\beta}'\mathbf{x}_i}}{\mathbf{s}^{(0)}(\boldsymbol{\beta};u)} + (1-a)\begin{bmatrix} \dfrac{x_{i1}e^{\beta_1 x_{i1}} - e_1(\beta_1;u)e^{\beta_1 x_{i1}}}{s_1^{(0)}(\beta_1;u)} \\ \vdots \\ \dfrac{x_{ip}e^{\beta_p x_{ip}} - e_p(\beta_p;u)e^{\beta_p x_{ip}}}{s_p^{(0)}(\beta_p;u)} \end{bmatrix} \right\} dF(u),$$

$N_i(u) = I(t_i \le u, \delta_i = 1)$ *and* $F(u) = E\{N_i(u)\}$. *Furthermore,* $\mathbf{w}_i^a(\boldsymbol{\beta})$ *has zero mean at* $\boldsymbol{\beta} = \boldsymbol{\beta}^*(a)$, *i.e.,* $E\{\mathbf{w}_i^a(\boldsymbol{\beta}^*(a))\} = \mathbf{0}$ for $a \in [0,1]$.

Lemma 2 (asymptotic linear expression)

$$\frac{1}{\sqrt{n}}\frac{dCV(a)}{da}=\dot{\mathbf{h}}(\boldsymbol{\beta}^*(a))'\mathbf{v}^a(\boldsymbol{\beta}^*(a))^{-1}\frac{1}{\sqrt{n}}\sum_{i=1}^{n}\mathbf{w}_i^1(\boldsymbol{\beta}^*(a))+o_P(1),$$

where $\dot{h}(\boldsymbol{\beta})$ *is defined to be* $\dot{h}(\boldsymbol{\beta})=\partial h(a,\boldsymbol{\beta})/\partial a$, *which does not depend on* a.

Proof of Lemma 1: Based on a similar calculation to Appendix of Lin and Wei (1989),

$$\begin{aligned}\frac{1}{\sqrt{n}}\mathbf{U}_n^a(\boldsymbol{\beta})&=\frac{1}{\sqrt{n}}\sum_{i=1}^{n}\int_0^\infty\{\mathbf{x}_i-\mathbf{E}^a(\boldsymbol{\beta};u)\}dN_i(u)\\&=\frac{1}{\sqrt{n}}\sum_{i=1}^{n}\int_0^\infty\{\mathbf{x}_i-\mathbf{e}^a(\boldsymbol{\beta};u)\}dN_i(u)-\sqrt{n}\int_0^\infty\{\mathbf{E}^a(\boldsymbol{\beta};u)-\mathbf{e}^a(\boldsymbol{\beta};u)\}dF(u)+o_P(1),\end{aligned}\tag{A.1}$$

where

$$\mathbf{E}^a(\boldsymbol{\beta};u)=a\mathbf{E}(\boldsymbol{\beta};u)+(1-a)\begin{bmatrix}E_1(\beta_1;u)\\ \vdots\\ E_p(\beta_p;u)\end{bmatrix},\ \mathbf{e}^a(\boldsymbol{\beta};u)=a\mathbf{e}(\boldsymbol{\beta};u)+(1-a)\begin{bmatrix}e_1(\beta_1;u)\\ \vdots\\ e_p(\beta_p;u)\end{bmatrix}.$$

Based on a Taylor expansion of $g(x,y)=x/y$ around (a,b), one has $x/y-a/b\approx x/b-ay/b^2$.

This leads to

$$\begin{aligned}\sqrt{n}\{E_j(\beta_j;u)-e_j(\beta_j;u)\}&=\sqrt{n}\{S_j^{(1)}(\beta_j;u)/S_j^{(0)}(\beta_j;u)-s_j^{(1)}(\beta_j;u)/s_j^{(0)}(\beta_j;u)\}\\&=\frac{1}{\sqrt{n}s_j^{(0)}(\beta_j;u)}\{S_j^{(1)}(\beta_j;u)-e_j(\beta_j;u)S_j^{(0)}(\beta_j;u)\}+o_P(1)\\&=\frac{1}{\sqrt{n}s_j^{(0)}(\beta_j;u)}\sum_{i=1}^{n}Y_i(u)\{x_{ij}e^{\beta_j x_{ij}}-e_j(\beta_j;u)e^{\beta_j x_{ij}}\}+o_P(1).\end{aligned}$$

Similarly,

$$\begin{aligned}\sqrt{n}\{\mathbf{E}(\boldsymbol{\beta};u)-\mathbf{e}(\boldsymbol{\beta};u)\}&=\sqrt{n}\{\mathbf{S}^{(1)}(\boldsymbol{\beta};u)/\mathbf{S}^{(0)}(\boldsymbol{\beta};u)-\mathbf{s}^{(1)}(\boldsymbol{\beta};u)/\mathbf{s}^{(0)}(\boldsymbol{\beta};u)\}\\&=\frac{1}{\sqrt{n}\mathbf{s}^{(0)}(\boldsymbol{\beta};u)}\{\mathbf{S}^{(1)}(\boldsymbol{\beta};u)-\mathbf{e}(\boldsymbol{\beta};u)\mathbf{S}^{(0)}(\boldsymbol{\beta};u)\}+o_P(1)\\&=\frac{1}{\sqrt{n}\mathbf{s}^{(0)}(\boldsymbol{\beta};u)}\sum_{i=1}^{n}Y_i(u)\{\mathbf{x}_i e^{\boldsymbol{\beta}'\mathbf{x}_i}-\mathbf{e}(\boldsymbol{\beta};u)e^{\boldsymbol{\beta}'\mathbf{x}_i}\}+o_P(1).\end{aligned}$$

Combining these results,

$$\sqrt{n}\{\,\mathbf{E}^a(\boldsymbol{\beta};u)-\mathbf{e}^a(\boldsymbol{\beta};u)\,\}=a\sqrt{n}\{\,\mathbf{E}(\boldsymbol{\beta};u)-\mathbf{e}(\boldsymbol{\beta};u)\,\}+(1-a)\sqrt{n}\begin{bmatrix} E_1(\beta_1;u)-e_1(\beta_1;u)\\ \vdots \\ E_p(\beta_p;u)-e_p(\beta_p;u)\end{bmatrix}$$

$$=\frac{1}{\sqrt{n}}\sum_{i=1}^{n}Y_i(u)\left\{a\frac{\mathbf{x}_i e^{\boldsymbol{\beta}'\mathbf{x}_i}-\mathbf{e}(\boldsymbol{\beta};u)e^{\boldsymbol{\beta}'\mathbf{x}_i}}{\mathbf{s}^{(0)}(\boldsymbol{\beta};u)}+(1-a)\begin{bmatrix}\dfrac{x_{i1}e^{\beta_1 x_{i1}}-e_1(\beta_1;u)e^{\beta_1 x_{i1}}}{s_1^{(0)}(\beta_1;u)}\\ \vdots \\ \dfrac{x_{ip}e^{\beta_p x_{ip}}-e_p(\beta_p;u)e^{\beta_p x_{ip}}}{s_p^{(0)}(\beta_p;u)}\end{bmatrix}\right\}+o_P(1)\,,$$

and thus

$$\sqrt{n}\int_0^\infty\{\mathbf{E}^a(\boldsymbol{\beta};u)-\mathbf{e}^a(\boldsymbol{\beta};u)\}dF(u)$$

$$=\frac{1}{\sqrt{n}}\sum_{i=1}^{n}\int_0^\infty Y_i(u)\left\{a\frac{\mathbf{x}_i e^{\boldsymbol{\beta}'\mathbf{x}_i}-\mathbf{e}(\boldsymbol{\beta};u)e^{\boldsymbol{\beta}'\mathbf{x}_i}}{\mathbf{s}^{(0)}(\boldsymbol{\beta};u)}+(1-a)\begin{bmatrix}\dfrac{x_{i1}e^{\beta_1 x_{i1}}-e_1(\beta_1;u)e^{\beta_1 x_{i1}}}{s_1^{(0)}(\beta_1;u)}\\ \vdots \\ \dfrac{x_{ip}e^{\beta_p x_{ip}}-e_p(\beta_p;u)e^{\beta_p x_{ip}}}{s_p^{(0)}(\beta_p;u)}\end{bmatrix}\right\}dF(u)+o_P(1)\,.$$

Hence by (A.1),

$$\frac{1}{\sqrt{n}}\mathbf{U}_n^a(\boldsymbol{\beta})=\frac{1}{\sqrt{n}}\sum_{i=1}^{n}\mathbf{w}_i^a(\boldsymbol{\beta})+o_P(1)\,.$$

It remains to show $E\{\mathbf{w}_i^a(\boldsymbol{\beta}^*(a))\}=\mathbf{0}$. One can show that for any $\boldsymbol{\beta}$

$$E\left[\int_0^\infty Y_i(u)\left\{a\frac{\mathbf{x}_i e^{\boldsymbol{\beta}'\mathbf{x}_i}-\mathbf{e}(\boldsymbol{\beta};u)e^{\boldsymbol{\beta}'\mathbf{x}_i}}{\mathbf{s}^{(0)}(\boldsymbol{\beta};u)}+(1-a)\begin{bmatrix}\dfrac{x_{i1}e^{\beta_1 x_{i1}}-e_1(\beta_1;u)e^{\beta_1 x_{i1}}}{s_1^{(0)}(\beta_1;u)}\\ \vdots \\ \dfrac{x_{ip}e^{\beta_p x_{ip}}-e_p(\beta_p;u)e^{\beta_p x_{ip}}}{s_p^{(0)}(\beta_p;u)}\end{bmatrix}\right\}dF(u)\right]$$

$$=\int_0^\infty\left\{a\frac{E[\,Y_i(u)\{\mathbf{x}_i e^{\boldsymbol{\beta}'\mathbf{x}_i}-\mathbf{e}(\boldsymbol{\beta};u)e^{\boldsymbol{\beta}'\mathbf{x}_i}\,\}\,]}{\mathbf{s}^{(0)}(\boldsymbol{\beta};u)}+(1-a)\begin{bmatrix}\dfrac{E[\,Y_i(u)\{\,x_{i1}e^{\beta_1 x_{i1}}-e_1(\beta_1;u)e^{\beta_1 x_{i1}}\,\}\,]}{s_1^{(0)}(\beta_1;u)}\\ \vdots \\ \dfrac{E[\,Y_i(u)\{\,x_{ip}e^{\beta_p x_{ip}}-e_p(\beta_p;u)e^{\beta_p x_{ip}}\,\}\,]}{s_p^{(0)}(\beta_p;u)}\end{bmatrix}\right\}dF(u)=\mathbf{0}.$$

By this and the fact that the martingale

$$M_i(t) = N_i(t) - \int_0^\infty Y_i(u) e^{\boldsymbol{\beta}_0' \mathbf{x}_i} h_0(u) du$$

has zero mean (Andersen et al., 1993),

$$E\{\mathbf{w}_i^a(\boldsymbol{\beta})\} = E\left[\int_0^\infty \{\mathbf{x}_i - \mathbf{e}^a(\boldsymbol{\beta};u)\} dN_i(u)\right] = \int_0^\infty E[\{\mathbf{x}_i - \mathbf{e}^a(\boldsymbol{\beta};u)\} Y_i(u) e^{\boldsymbol{\beta}_0' \mathbf{x}_i} h_0(u)] du$$
$$= \mathbf{h}(a, \boldsymbol{\beta}).$$

Since $\boldsymbol{\beta}^*(a)$ is a solution to $\mathbf{h}(a, \boldsymbol{\beta}) = 0$, $E\{\mathbf{w}_i^a(\boldsymbol{\beta}^*(a))\} = \mathbf{0}$ for $a \in [0,1]$. □

Proof of Lemma 2: The derivative of $\mathbf{0} = \mathbf{U}_{n,(-k)}^a(\hat{\boldsymbol{\beta}}_{(-k)}(a))$ with respect to a leads to

$$d\hat{\boldsymbol{\beta}}_{(-k)}(a)/da = \{\mathbf{V}_{n,(-k)}^a(\hat{\boldsymbol{\beta}}_{(-k)}(a))\}^{-1} \dot{\mathbf{U}}_{n,(-k)}^a(\hat{\boldsymbol{\beta}}_{(-k)}(a)),$$

where $\dot{\mathbf{U}}_{n,(-k)}^a(\boldsymbol{\beta}) = d\mathbf{U}_{n,(-k)}^a(\boldsymbol{\beta})/da$. Then one has

$$dCV(a)/da = \sum_{k=1}^K \dot{\mathbf{U}}_{n,(-k)}^a(\hat{\boldsymbol{\beta}}_{(-k)}(a))' \{\, \mathbf{V}_{n,(-k)}^a(\hat{\boldsymbol{\beta}}_{(-k)}(a)) \,\}^{-1} \{\, \mathbf{U}_n^1(\hat{\boldsymbol{\beta}}_{(-k)}(a)) - \mathbf{U}_{n,(-k)}^1(\hat{\boldsymbol{\beta}}_{(-k)}(a)) \,\},$$

It follows from martingale calculus that $n^{-1}\dot{\mathbf{U}}_{n,(-k)}^a(\boldsymbol{\beta}) = \dot{\mathbf{h}}(\boldsymbol{\beta}) + o_P(1)$, where

$$\dot{h}(\boldsymbol{\beta}) = \frac{\partial h(a, \boldsymbol{\beta})}{\partial a} = \int_0^\infty \left\{ -\mathbf{e}(\boldsymbol{\beta};u) + \begin{bmatrix} e_1(\beta_1;u) \\ \vdots \\ e_p(\beta_p;u) \end{bmatrix} \right\} s^{(0)}(\boldsymbol{\beta}_0;u) h_0(u) du.$$

Since $\hat{\boldsymbol{\beta}}_{(-k)}(a)$ converges in probability to $\boldsymbol{\beta}^*(a)$,

$$n^{-1}\dot{\mathbf{U}}_{n,(-k)}^a(\hat{\boldsymbol{\beta}}_{(-k)}(a)) = \dot{\mathbf{h}}(\boldsymbol{\beta}^*(a)) + o_P(1), \quad n^{-1}\mathbf{V}_{n,(-k)}^a(\hat{\boldsymbol{\beta}}_{(-k)}(a)) = \mathbf{v}^a(\boldsymbol{\beta}^*(a)) + o_P(1).$$

These equations imply

$$\dot{\mathbf{U}}_{n,(-k)}^a(\hat{\boldsymbol{\beta}}_{(-k)}(a))' \{\mathbf{V}_{n,(-k)}^a(\hat{\boldsymbol{\beta}}_{(-k)}(a))\}^{-1} = \dot{\mathbf{h}}(\boldsymbol{\beta}^*(a))' \mathbf{v}^a(\boldsymbol{\beta}^*(a))^{-1} + o_P(1),$$

where we suppose that $\mathbf{v}^a(\boldsymbol{\beta}^*(a))$ is positive definite. By Lemma 1,

$$\mathbf{U}_n^1(\hat{\boldsymbol{\beta}}_{(-k)}(a)) - \mathbf{U}_{n,(-k)}^1(\hat{\boldsymbol{\beta}}_{(-k)}(a)) = \sum_{i\in\Im_k}\mathbf{w}_i^1(\boldsymbol{\beta}^*(a)) + o_P(\sqrt{n})\,.$$

Applying the preceding two equations to $dCV(a)/da$, we get the desired results.□

Proof of Proposition 1: Lemma 2 implies

$$\frac{1}{n}\frac{dCV(a)}{da} = cv(a) + o_P\left(\frac{1}{\sqrt{n}}\right).$$

where $cv(a) = \dot{\mathbf{h}}(\boldsymbol{\beta}^*(a))'\mathbf{v}^a(\boldsymbol{\beta}^*(a))^{-1}E\{\mathbf{w}_i^1(\boldsymbol{\beta}^*(a))\}$. Since $dCV(a)/da\,|_{a=\hat{a}} = 0$, $\hat{a}$ converges in probability to a solution to $cv(a) = 0$. From the formula of $\dot{h}(\boldsymbol{\beta})$, as shown before, one can verify $\dot{h}(\boldsymbol{\beta}) \neq \mathbf{0}$ for any $\boldsymbol{\beta}$. Hence, for $cv(a) = 0$ to be hold, it must satisfy $E\{\mathbf{w}_i^1(\boldsymbol{\beta}^*(a))\} = \mathbf{0}$. By Lemma 1, this hold when $a = 1$. Hence, $\hat{a}$ converges in probability to $a = 1$.

To prove that $\hat{\boldsymbol{\beta}}(\hat{a})$ converges in probability to $\boldsymbol{\beta}_0$, we note that

$$\|\hat{\boldsymbol{\beta}}(\hat{a}) - \boldsymbol{\beta}_0\| \le \|\hat{\boldsymbol{\beta}}(\hat{a}) - \hat{\boldsymbol{\beta}}(1)\| + \|\hat{\boldsymbol{\beta}}(1) - \boldsymbol{\beta}^*(1)\|\,.$$

Since $\hat{\boldsymbol{\beta}}(1)$ is the usual partial likelihood estimator, $\|\hat{\boldsymbol{\beta}}(1) - \boldsymbol{\beta}^*(1)\| = o_P(1)$. It remains to show that $\|\hat{\boldsymbol{\beta}}(\hat{a}) - \hat{\boldsymbol{\beta}}(1)\| = o_P(1)$. By differentiating $0 = \mathbf{U}_n^a(\hat{\boldsymbol{\beta}}(a))$ with respect to a, we have $d\hat{\boldsymbol{\beta}}(a)/da = \mathbf{V}_n^a(\hat{\boldsymbol{\beta}}(a))^{-1}\dot{\mathbf{U}}_n^a(\hat{\boldsymbol{\beta}}(a))$, where $\dot{\mathbf{U}}_n^a(\boldsymbol{\beta}) = d\mathbf{U}_n^a(\boldsymbol{\beta})/da$. Hence,

$$\hat{\boldsymbol{\beta}}(\hat{a}) - \hat{\boldsymbol{\beta}}(1) = \mathbf{V}_n^1(\hat{\boldsymbol{\beta}}(1))^{-1}\dot{\mathbf{U}}_n^1(\hat{\boldsymbol{\beta}}(1))(\hat{a}-1) + o_P(|\hat{a}-1|)\,. \qquad \text{(A.2)}$$

Since $n^{-1}\dot{\mathbf{U}}_n^1(\hat{\boldsymbol{\beta}}(1)) = \dot{\mathbf{h}}(\boldsymbol{\beta}_0) + o_P(1)$ and $n^{-1}\mathbf{V}_n^a(\hat{\boldsymbol{\beta}}(1)) = \mathbf{v}^a(\boldsymbol{\beta}_0) + o_P(1)$, $\mathbf{V}_n^1(\hat{\boldsymbol{\beta}}(1))^{-1}\dot{\mathbf{U}}_n^1(\hat{\boldsymbol{\beta}}(1))$ has order $O_P(1)$. Thus, $\|\hat{\boldsymbol{\beta}}(\hat{a}) - \hat{\boldsymbol{\beta}}(1)\| = O_P(|\hat{a}-1|) = o_P(1)$.□

Proof of Proposition 2: Let $z_i(\boldsymbol{\beta}_0) = -\partial\dot{\mathbf{h}}(\boldsymbol{\beta}^*(a))'\mathbf{v}^a(\boldsymbol{\beta}^*(a))^{-1}\mathbf{w}_i^1(\boldsymbol{\beta}^*(a))/\partial a\,|_{a=1}$. Then,

$$-\frac{1}{n}\frac{d^2CV(a)}{da^2}\bigg|_{a=1} = E\{z_1(\boldsymbol{\beta}_0)\} + o_P(1).$$

Applying a Taylor expansion to Lemma 2, we get

$$\sqrt{n}(\hat{a}-1) = \frac{\dot{\mathbf{h}}(\boldsymbol{\beta}_0)'\mathbf{v}^1(\boldsymbol{\beta}_0)^{-1}}{E\{z_1(\boldsymbol{\beta}_0)\}}\frac{1}{\sqrt{n}}\sum_{i=1}^{n}\mathbf{w}_i^1(\boldsymbol{\beta}_0) + o_P(1) \qquad \text{(A.3)}$$

By the definition of $\mathbf{w}_i^a(\boldsymbol{\beta})$ in Lemma 1,

$$\mathbf{w}_i^1(\boldsymbol{\beta}) = \int_0^\infty \{\mathbf{x}_i - \mathbf{e}(\boldsymbol{\beta};u)\}dM_i(u),$$

which has zero mean. Also, by the martingale calculus (Andersen et al., 1993),

$$E\{\mathbf{w}_i^1(\boldsymbol{\beta}_0)^{\otimes 2}\} = E\left[\int_0^\infty \{\mathbf{x}_i - \mathbf{e}(\boldsymbol{\beta};u)\}^{\otimes 2} Y_i(u) e^{\boldsymbol{\beta}_0'\mathbf{x}_i} h_0(u)du\right]$$
$$= \mathbf{v}^1(\boldsymbol{\beta}_0).$$

From the central limit theorem, the right-side of equation converges weakly to a normal distribution with mean zero and variance

$$v_{CV}(\boldsymbol{\beta}_0) = \frac{\dot{\mathbf{h}}(\boldsymbol{\beta}_0)'\mathbf{v}^1(\boldsymbol{\beta}_0)^{-1}\dot{\mathbf{h}}(\boldsymbol{\beta}_0)}{[E\{z_i(\boldsymbol{\beta}_0)\}]^2}.$$

Now we prove the second part of Proposition 2. By a Taylor expansion and Lemma 1,

$$\sqrt{n}(\hat{\boldsymbol{\beta}}(1) - \boldsymbol{\beta}_0) = \mathbf{v}^1(\boldsymbol{\beta}_0)^{-1}\frac{1}{\sqrt{n}}\sum_{i=1}^{n}\mathbf{w}_i^1(\boldsymbol{\beta}_0) + o_P(1).$$

By (A.2) and (A.3), we have

$$\sqrt{n}(\hat{\boldsymbol{\beta}}(\hat{a}) - \hat{\boldsymbol{\beta}}(1)) = \mathbf{v}^1(\boldsymbol{\beta}_0)^{-1}\dot{\mathbf{h}}(\boldsymbol{\beta}_0)\sqrt{n}(\hat{a}-1) + o_P(1)$$
$$= \frac{\mathbf{v}^1(\boldsymbol{\beta}_0)^{-1}\dot{\mathbf{h}}(\boldsymbol{\beta}_0)\dot{\mathbf{h}}(\boldsymbol{\beta}_0)'\mathbf{v}^1(\boldsymbol{\beta}_0)^{-1}}{E\{z_1(\boldsymbol{\beta}_0)\}}\frac{1}{\sqrt{n}}\sum_{i=1}^{n}\mathbf{w}_i^1(\boldsymbol{\beta}_0) + o_P(1).$$

The preceding two equations yield

$$\sqrt{n}(\hat{\boldsymbol\beta}(\hat a)-\boldsymbol\beta_0)=\left(\frac{\mathbf{v}^1(\boldsymbol\beta_0)^{-1}\dot{\mathbf{h}}(\boldsymbol\beta_0)\dot{\mathbf{h}}(\boldsymbol\beta_0)'}{E\{z_1(\boldsymbol\beta_0)\}}+\mathbf{I}_p\right)\mathbf{v}^1(\boldsymbol\beta_0)^{-1}\frac{1}{\sqrt{n}}\sum_{i=1}^n\mathbf{w}_i^1(\boldsymbol\beta_0)+o_P(1).$$

Hence, $\sqrt{n}(\hat{\boldsymbol\beta}(\hat a)-\boldsymbol\beta_0)$ converges weakly to a normal distribution with mean zero and covariance matrix

$$\boldsymbol\Sigma(\boldsymbol\beta_0)=\left(\frac{\mathbf{v}^1(\boldsymbol\beta_0)^{-1}\dot{\mathbf{h}}(\boldsymbol\beta_0)\dot{\mathbf{h}}(\boldsymbol\beta_0)'}{E\{z_1(\boldsymbol\beta_0)\}}+\mathbf{I}_p\right)\mathbf{v}^1(\boldsymbol\beta_0)^{-1}\left(\frac{\mathbf{v}^1(\boldsymbol\beta_0)^{-1}\dot{\mathbf{h}}(\boldsymbol\beta_0)\dot{\mathbf{h}}(\boldsymbol\beta_0)'}{E\{z_1(\boldsymbol\beta_0)\}}+\mathbf{I}_p\right)'.\square$$

Variance estimation

The asymptotic variance $\boldsymbol\Sigma(\boldsymbol\beta_0)$ can be consistently estimated by $\boldsymbol\Sigma_n^{\hat a}(\hat{\boldsymbol\beta}(\hat a))$, where

$$\boldsymbol\Sigma_n^a(\boldsymbol\beta)=\mathbf{A}_n^a(\boldsymbol\beta)\left\{\frac{\mathbf{V}_n^a(\boldsymbol\beta)}{n}\right\}^{-1}\mathbf{A}_n^a(\boldsymbol\beta)',\quad \mathbf{A}_n^a(\boldsymbol\beta)=\frac{\mathbf{V}_n^a(\boldsymbol\beta_0)^{-1}\dot{\mathbf{h}}_n(\boldsymbol\beta)\dot{\mathbf{h}}_n(\boldsymbol\beta)'}{-d^2CV(a)/da^2}+\mathbf{I}_p,$$

$$\dot{h}_n(\boldsymbol\beta)=\frac{\partial\mathbf{U}_n^a(\boldsymbol\beta)}{\partial a}=\sum_{i=1}^n\delta_i\{-\mathbf{E}(\boldsymbol\beta;t_i)+(E_1(\beta_1;t_i),\ldots,E_p(\beta_p;t_i))'\}.$$

This suggests the following approximation

$$Cov(\hat{\boldsymbol\beta}(\hat a))\approx\boldsymbol\Sigma_n^{\hat a}(\hat{\boldsymbol\beta}(\hat a))/n. \tag{A.4}$$

Additional simulation results

We examine the usefulness of the approximation (A.4) via simulations. The simulation setups are the same as the main article. The results under scenario 1 are summarized in Table A. In general, the average of the estimated standard deviation gives reasonable approximation to the sample standard deviation of the regression estimators.

Table A: *Simulation results with n=100 and p=100 based on 50 replications.*

Sparse cases	$\boldsymbol{\beta}=(1.5,1.5,\underbrace{0,\ldots,0}_{\times 98})$			$\boldsymbol{\beta}=(1,1,-1,-1,\underbrace{0,\ldots,0}_{\times 96})$		
	$E(\hat{\beta}_j)$	$SD(\hat{\beta}_j)$	$ESD(\hat{\beta}_j)$	$E(\hat{\beta}_j)$	$SD(\hat{\beta}_j)$	$ESD(\hat{\beta}_j)$
$\beta_p=0$	-0.023	0.158	0.167	0.005	0.185	0.169
$\beta_1=1.5$ or 1	0.966	0.195	0.199	0.554	0.146	0.180
Sparse cases	$\boldsymbol{\beta}=(\underbrace{0.8,\ldots,0.8}_{\times 5},\underbrace{0,\ldots,0}_{\times 95})$			$\boldsymbol{\beta}=(\underbrace{0.4,\ldots,0.4}_{\times 5},\underbrace{-0.4,\ldots,-0.4}_{\times 5},\underbrace{0,\ldots,0}_{\times 90})$		
	$E(\hat{\beta}_j)$	$SD(\hat{\beta}_j)$	$ESD(\hat{\beta}_j)$	$E(\hat{\beta}_j)$	$SD(\hat{\beta}_j)$	$ESD(\hat{\beta}_j)$
$\beta_p=0$	-0.036	0.148	0.173	0.057	0.173	0.178
$\beta_1=0.8$ or 0.4	0.489	0.141	0.192	0.270	0.164	0.179
Less sparse cases	$\boldsymbol{\beta}=(\underbrace{0.4,\ldots,0.4}_{\times 10},\underbrace{0,\ldots,0}_{\times 90})$			$\boldsymbol{\beta}=(\underbrace{0.2,\ldots,0.2}_{\times 10},\underbrace{-0.2,\ldots,-0.2}_{\times 10},\underbrace{0,\ldots,0}_{\times 80})$		
	$E(\hat{\beta}_j)$	$SD(\hat{\beta}_j)$	$ESD(\hat{\beta}_j)$	$E(\hat{\beta}_j)$	$SD(\hat{\beta}_j)$	$ESD(\hat{\beta}_j)$
$\beta_p=0$	0.018	0.171	0.183	-0.022	0.152	0.170
$\beta_1=0.4$ or 0.2	0.299	0.147	0.190	0.152	0.173	0.176
Less sparse cases	$\boldsymbol{\beta}=(\underbrace{0.2,\ldots,0.2}_{\times 15},\underbrace{0,\ldots,0}_{\times 85})$			$\boldsymbol{\beta}=(\underbrace{0.1,\ldots,0.1}_{\times 15},\underbrace{-0.1,\ldots,-0.1}_{\times 15},\underbrace{0,\ldots,0}_{\times 70})$		
	$E(\hat{\beta}_j)$	$SD(\hat{\beta}_j)$	$ESD(\hat{\beta}_j)$	$E(\hat{\beta}_j)$	$SD(\hat{\beta}_j)$	$ESD(\hat{\beta}_j)$
$\beta_p=0$	-0.008	0.184	0.167	0.058	0.172	0.176
$\beta_1=0.2$ or 0.1	0.143	0.137	0.168	0.073	0.165	0.178

REFERENCES

Andersen, P. K., Borgan, O., Gill, R. D., and Keiding, N. (1993), *Statistical Models Based on Counting Processes*. New York: Springer-Verlag.

Lin, D. Y. and Wei, L. J. (1989). The robust inference for the Cox proportional hazards model. *Journal of the American Statistical Association* ***84***, 1074-1078.

Struthers, C. A. and Kalbfleish, J. D. (1986). Misspecified proportional hazard models, *Biometrika* **73**, 363-369.
